# A Model of Glucocorticoid Receptor Interaction With Coregulators Predicts Transcriptional Regulation of Target Genes

**DOI:** 10.3389/fphar.2019.00214

**Published:** 2019-03-13

**Authors:** Federico Monczor, Antonia Chatzopoulou, Carlos Daniel Zappia, René Houtman, Onno C. Meijer, Carlos P. Fitzsimons

**Affiliations:** ^1^Laboratorio de Farmacología de Receptores, Instituto de Investigaciones Farmacológicas, Universidad de Buenos Aires–Consejo Nacional de Investigaciones Científicas y Técnicas, Buenos Aires, Argentina; ^2^Leiden Academic Center for Drug Research, Leiden University, Leiden, Netherlands; ^3^PamGene International B.V., ′s-Hertogenbosch, Netherlands; ^4^Division of Endocrinology, Department of Internal Medicine, Leiden University Medical Centre, Leiden, Netherlands; ^5^Neuroscience Collaboration, Swammerdam Institute for Life Sciences, University of Amsterdam, Amsterdam, Netherlands

**Keywords:** transcriptional activity, cofactor interaction, nuclear receptor, receptor allosterism, glucocorticoid receptor

## Abstract

Regulatory factors that control gene transcription in multicellular organisms are assembled in multicomponent complexes by combinatorial interactions. In this context, nuclear receptors provide well-characterized and physiologically relevant systems to study ligand-induced transcription resulting from the integration of cellular and genomic information in a cell- and gene-specific manner. Here, we developed a mathematical model describing the interactions between the glucocorticoid receptor (GR) and other components of a multifactorial regulatory complex controlling the transcription of GR-target genes, such as coregulator peptides. We support the validity of the model in relation to gene-specific GR transactivation with gene transcription data from A549 cells and *in vitro* real time quantification of coregulator-GR interactions. The model accurately describes and helps to interpret ligand-specific and gene-specific transcriptional regulation by the GR. The comprehensive character of the model allows future insight into the function and relative contribution of the molecular species proposed in ligand- and gene-specific transcriptional regulation.

## Introduction

Glucocorticoids contribute to the maintenance of homeostasis in almost all organs and tissues under basal and stress conditions in higher organisms. Many of these homeostatic functions are exerted directly by GC binding to the GR, which regulates the transcription of broad networks of target genes. The GR is a ligand-induced transcription factor that, upon GC binding, translocates to the nucleus and promotes the assembly of multiprotein regulatory complexes at genomic GREs (Darimont et al., 1998; Rogatsky et al., 2003; Luecke and Yamamoto, 2005).

A commonly accepted framework of nuclear receptor function is that ligand binding to the receptor induces the formation of a MTC that includes coregulator proteins and hormone response elements on the DNA. However, this framework falls short in explaining how nuclear receptor binding to similar hormone response elements is able to differentially regulate individual genes in a given cell environment (Bain et al., 2014). Currently accepted theoretical models of ligand-induced nuclear receptor-mediated gene expression predict that the amount of gene expression depends on the amount of ligand-activated receptor species present in the system (Kenakin, 2004; Chow et al., 2011). Therefore, the properties of the ligand dose-gene response curve provide a quantitative means to investigate gene expression. However, the cellular response to GR stimulation is not always a simple binary response to GC binding (John et al., 2009) and numerous factors contribute to GC action at each step of the GR signaling cascade from ligand binding to its end-point result, the induction of gene transcription. Importantly, these factors appear to be tissue-, cell type-, and gene promoter-specific (Rogatsky et al., 2003; Bolton et al., 2007; So et al., 2007; Oakley and Cidlowski, 2011; Gertz et al., 2013).

The intermediate steps between ligand binding to the GR and the resulting gene promoter-specific induction include ligand binding and interaction with coregulator proteins that promote (coactivators) or inhibit (corepressors) gene expression and DNA binding (Meijer, 2002). Ligand binding *per se* is an important factor, as different ligands can induce specific GR interaction patterns with coregulators (Coghlan et al., 2003; Zalachoras et al., 2013). Further, specific coregulator binding to the activated GR plays a role in gene-specific induction (Lachize et al., 2009). Coregulators bind to the GR and modulate its transcriptional activity by modifying DNA structure. In particular, recruitment of transcriptional coactivators may destabilize chromatin by specific mechanisms including histone acetylation and contacts with the basal transcriptional machinery. In contrast, the recruitment of corepressors may stabilize chromatin by targeting histone deacetylases (Collingwood et al., 1999). Similarly, recent studies have demonstrated that coregulators can behave as allosteric modulators of the GR, affecting ligand interactions (Pfaff and Fletterick, 2010). These observations highlight the complexity of the molecular interactions taking place during the formation of a MTC.

Here, we aim to develop a model of GR interaction with coregulators and other components of a MTC that could describe and interpret differential ligand-specific transcriptional regulation of individual target genes. Our model is conceptually based on the cubic ternary complex receptor–occupancy model (Weiss et al., 1996), which has been extensively used before to describe the pharmacological behavior of multifactor complexes containing receptor proteins (Monczor et al., 2003; Fitzsimons et al., 2004; Tubio et al., 2010; Granja-Galeano et al., 2017). To experimentally test the model, we focused on gene transactivation, where the GR interacts with cofactors and DNA rather than acting by tethering mechanisms based on protein:protein interactions that influence the activity of other transcription factors without directly contacting the DNA (Newton and Holden, 2007). We used a set of previously identified GR-responsive genes in A549 human lung adenocarcinoma cells (Wang et al., 2004) and a MARCoNI. This array contained 54 coregulator-derived peptides representing nuclear receptor (NR)-boxes, or LXXLL motifs (Heery et al., 1997; Darimont et al., 1998), that interact with the activation function domain 2 (AF2) within the ligand-binding domain LBD of NRs. This assay can be used as a sensor for receptor conformation and activity status and allows the characterization of nuclear receptor binding to coregulators (Hur et al., 2004; Moore et al., 2004; Wu et al., 2004; Estébanez-Perpiñá et al., 2005; Teichert et al., 2009).

We found significant GR ligand-dependent differences in the relative efficacy and potency of induction of three GR-responsive genes in A549 cells and in the binding of coregulators to GR. Based on the behavior of GR ligands in A549 cells and *in vitro* coregulator recruitment, we developed a model of transcriptional regulation by the GR including ligand binding and interaction with coregulators that interprets gene-induction potency observed in living cells. The model includes a unique parameter δ describing the allosteric interaction between the components of the multifactorial complex. Using the non-independent action indicated by the model, we were able to interpret gene-specific transcriptional inhibition by partial agonists in the presence of a full agonist of the GR in A549 cells. This model is supported by previous observations on GR-mediated gene-specific transcriptional activation and could be used to understand and interpret the pharmacological action of ligands that selectively modulate GR-dependent transcriptional activity.

## Materials and Methods

### Cell Culture and Treatments

A549 human lung carcinoma cells were obtained from the American Type Culture Collection (ATCC: Manassas, VA, United States) and cultured in complete medium (DMEM with 14.5 g/L glucose, supplemented with 10% fetal bovine serum and 1% penicillin and streptomycin, all from Invitrogen). Cells were cultured at 37°C in a humidified 5% CO_2_ atmosphere. For cell passaging or plating, cells were first washed out with 1X phosphate buffered saline (Invitrogen) and then trypsinized using 1X trypsin-EDTA (10X 0.5% Trypsin, Invitrogen). For all ligand treatments, A549 cells (200,000 cells/well) were seeded into six-well plates (Corning International, NY, United States) and cultured for 24 h in complete medium and maintained in steroid-free medium prepared as complete medium but using charcoal-stripped serum (Adams et al., 2003) for 24 h before ligand or vehicle (ethanol) treatments. DEX, RU486, and CYP (Sigma) stock solutions (1 mM) were dissolved in 100% ethanol and kept at −20°C and further diluted in steroid-free medium before usage.

### RNA Isolation and cDNA Synthesis

Total cellular RNA was extracted using the TRIzol^®^ reagent (Invitrogen) following the supplier’s manual (Invitrogen). Total RNA was dissolved in RNAase free water, denatured for 5 min at 65°C and RNA concentration was quantified by spectrophotometric OD260 measurement using the Bioanalyzer (Agilent Technologies, Palo Alto, CA, United States). RNA samples were stored at −80°C until further use. 1 μg of total RNA was used for cDNA synthesis. In order to remove genomic DNA carry-over, RNA samples were treated with 1.5 u of DNAase I (Invitrogen) for 15 min at 25°C. DNAase I treated samples were then incubated at 65°C for 10 min following addition of 25 nM of EDTA (Invitrogen). Finally, they were reverse transcribed using the iSCRIPT^TM^ cDNA Synthesis Kit according to the manufacturer’s instructions (Bio-Rad). From each DNAase I treated RNA sample, a non-reverse transcribed (-RT) sample was similarly generated (reverse transcriptase was replaced with water). cDNA as well as –RT samples were kept at −20°C.

### Quantitative Polymerase Chain Reaction (qPCR)

Forward and reverse primer pairs against reference (b-actin) and GR were generated using the primer3 Input on line software^1^ and designs were based on publicly available human mRNA sequences. Primers were designed to have approximately 50% G/C content and to generate 150–250 bp amplicons. Primer pair specificity against target sequence was checked in the NCBI GenBank database using BLAST^2^. The sequences of the primers used to detect glucocorticoid-induced leucine zipper (GILZ), Solute Carrier 19A (SLC19A), and thrombomodulin (THBD) were provided by Dr. J. C. Wang and have been used before to detect gene expression in A549 cells (Wang et al., 2004). The sequences of the primers used to detect secretory leukocyte protease inhibitor (SLPI) and nuclear receptor subfamily 0 group B member 1 (NR0B1) were as follows: SLPI forward 5′-TCAAATGCCTGGATCCTGTTGA-3′; SLPI reverse 5′-GCATCAAACATTGGCCATAAGTC-3′; NR0B1 forward 5′-TGCTCTTTAACCCGGACGTG-3′; NR0B1 reverse 5′-GCGTCATCCTGGTGTGTTCA-3′. In all cases, primers were supplied by Isogen Life Sciences (Netherlands) and dissolved in water according to the supplier’s instructions and kept at −20°C until use.

qPCR monitoring and analysis was performed using the LightCycler^®^ Carousel-Based Detection System 2.0 (Roche). PCR reactions were performed in a total volume of 10 μl containing 2 μl of LightCycler^®^ FastStart DNA Master^PLUS^ SYBR Green I master mix (Roche), 2 μl undiluted cDNA and 1 μl of each forward (5 pmol/μl) and reverse primer (5 pmol/μl). Every PCR reaction mix was filled in the LightCycler glass capillaries which were subsequently closed and centrifuged using the LC Carousel Centrifuge 2.0 (Roche). Cycling conditions were a single pre-incubation step at 95°C for 10 min followed by 45 cycles of 10 s at 95°C, 10 s at 60°C and 10 s at 72°C. To verify that the primer pairs used yielded single PCR products, a dissociation protocol was added after thermocycling, determining dissociation of the PCR products from 65 to 95°C for 15 s. Finally, a cooling step was set for 20 s at 40°C.

To estimate the efficiency of the amplification reaction, serial half logarithm unit dilutions of cDNA from the A549 cells were used and standard curves were generated. The linear slope of the standard curve for each primer pair was estimated using GraphPad Prism 4 software and the efficiency was calculated based on the following equation (1).

(1) $$\text{Efficiency}=10^{-(1/\text{slope})}$$

Additionally, the -RT samples and a water-template were included in the analysis to confirm the absence of any residual DNA or contamination. All cDNA samples were analyzed in triplicates. Finally, the following equation (2) was used to calculate the fold induction of gene expression.

(2) $$\mathrm{Fold induction}=\frac{{\mathrm{Efficiency of target gene}}_{\mathrm{target}}^{\Delta \mathrm{cp}}{}^{\left(\mathrm{control}-\mathrm{experimental group}\right)}}{{\mathrm{Efficiency of reference gene}}_{\mathrm{reference}}^{\Delta \mathrm{cp}}{}^{\left(\mathrm{control}-\mathrm{experimental group}\right)}}$$

### Cell Transfection With siRNA

Transfections with siRNAs against the GR were performed as described before (Fitzsimons et al., 2013). Briefly, a total of 100 pmol of a GR targeting (Hs_NR3C1_6_HP validated siRNA, Qiagen and siGENOME NR0B1 siRNA, Dharmacon) or a non-targeting control siRNA (AllStrars Neg. siRNA AF 546, Qiagen or siGENOME Non-Targeting Control siRNAs #1, Dharmacon) were transfected into A549 cells using the Nucleofector I (Lonza) and the Cell Line Nucleofector^®^ Kit T for the A549 cell line, according to the supplier’s instructions (Lonza) using the U-29 Nucleofector program. The control siRNA was tagged with a red fluorophore, to monitor transfection efficacy, which was always higher than 90%. The cell medium was refreshed 1 day after transfection and all ligand treatments were performed 3 days after transfection.

### Western Blotting

Total cellular proteins were extracted from A549 cells with fresh RIPA lysis buffer (200 μl/well) on ice, transferred into 1.5 ml eppendorfs, mixed and kept at −20°C until use. Total protein concentration was measured using the BCA^TM^ Protein Assay Kit according to the supplier’s guidelines (Pierce). 3X of sample buffer was added to 10 μg of total protein, denatured for 5 min at 95°C, spinned shortly and then loaded onto 10% SDS-polyacrylamide gels. Samples were run through stacking gel at 100 V for 10 min and separating gel at 200 V for approximately 1 h. Protein transfer onto methanol-activated Immobilon^TM^ – P^SQ^ Transfer membranes (Millipore) was performed overnight at 4°C at 125 mA.

For the detection of the GR protein levels, the blots were incubated in blocking buffer, consisted of 5% low fat milk powder in TBST solution for 1 h at RT (10 ml/membrane) and subsequently with a primary antibody against the GR (H-300 rabbit polyclonal IgG, Santa Cruz), or against NR0B1 [Anti-NR0B1/Dax1 antibody (EP13786) – N-terminal (ab196649), abcam], or against alpha-tubulin antibody (clone DM1A, Sigma), or against GAPDH antibody (Santa Cruz). Primary antibodies were added in blocking buffer (1:2000 and 1:1000 dilution, respectively) for 1 h at RT (5 ml/membrane). Following 3X washing with TBST, the blots were probed with species-specific horseradish peroxidase-conjugated secondary antibodies (Santa Cruz) in blocking buffer (1:5000 dilution) for another 1 h at RT (10 ml/membrane) and finally washed 5X with TBST. For all incubations and washes rolling shakers were used. For luminescent signal detection, membranes were incubated with 10 ml of luminol solution, supplemented with 100 μl of enhancer solution and 3.1 μl of 30% H_2_O_2_ for approximately 1 min at RT in the dark. Following film exposure, development and fixation, GR protein levels among samples were quantified relatively to a-tubulin signal using the ImageJ software^3^ (Rasband, W.S., ImageJ, United States National Institutes of Health, Bethesda, MD, United States, 1997–2012).

### Peptide Interaction Profiling

Interactions between the GR-LBD and coregulator NR-box peptides were determined using a MARCoNI assay (PamChip no. 88011; PamGene International) as described before (Koppen et al., 2009; Zalachoras et al., 2013). Each array was incubated with a reaction mixture of 1 nM Purified Glucocorticoid Receptor Recombinant Human Protein, Ligand Binding Domain, (Thermo Fisher Scientific, cat # A15668), ALEXA488-conjugated anti-GST antibody and buffer F (PV4689, A-11131, and PV4547; Invitrogen). For ligand induced peptide interaction profiling experiments 1 μM DEX, RU486, CYP or solvent (2% DMSO in water) were added. Incubation was performed at 20°C in a PamStation96 (PamGene International). GR binding to each peptide on the array, reflected by fluorescent signal, was quantified by image analysis using BioNavigator software (PamGene International).

### Nuclear Translocation Assay

GR translocation to the nucleus was studied using a YFP-GR construct kindly supplied by Dr. Cidlowski (National Institute of Environmental Health Sciences, National Institutes of Health) as previously described, with some modifications (Fitzsimons et al., 2008). Briefly, the previously described protocol was scaled down to a 96-wells plate format and semi-automated. 6000 A549 cells/well were plated 24 h prior to transfection. The cells were transfected with YFP-GR plasmid using a Nuclefector I (Lonza) as described before. Complete medium was refreshed 24 h after transfection and 48-h after transfection cells were incubated for 6 h in steroid free medium. After this procedure, the GR localized to the cytosol in all cells as described before (Fitzsimons et al., 2008). The test compounds or vehicle (ethanol) were manually dispensed into the corresponding wells and cells were incubated with the compounds for 30 min. Subsequently, cells were fixed with 80% acetone in water and stained with Hoechst 3342 (1:10.000) for nuclear staining. All experiments were performed in triplicates. Three non-overlapping images were taken from each well by using a Zeiss Axiovert 200/200M inverted microscope and 10× magnification. DAPI and FITC filters with excitation wavelength 409 and 487 nm were used to excite Hoechst (blue emission) and YFP (green emission), respectively. All images were collected with the same settings in Microsoft Window’s BMP format. For post-acquisition image analysis Images were opened in ImageJ software and image backgrounds were subtracted by using the built in subtract background command with Rolling ball radius parameter set at 50. Subsequently, the images were opened with the CellProfiler software^4^ to automatically identify the nuclear and cytoplasm compartments of cells and the green fluorescence intensity in each compartment (Carpenter et al., 2006).

### Statistical Analysis

For comparisons between groups a two-tailed Student’s *t*-test was applied using GraphPad’s Prism 5 Software. For multigroup comparisons, a one-way ANOVA test with a Tukey’s post test was performed using the same software package. Dose response curves were fit to a sigmoidal (four-parameter logistic) curve using GraphPad’s Prism 5.

## Results

### Dexamethasone (DEX) and RU486 Induce the Expression of GR Responsive Genes With Different Pharmacological Parameters

**FIGURE 1:**
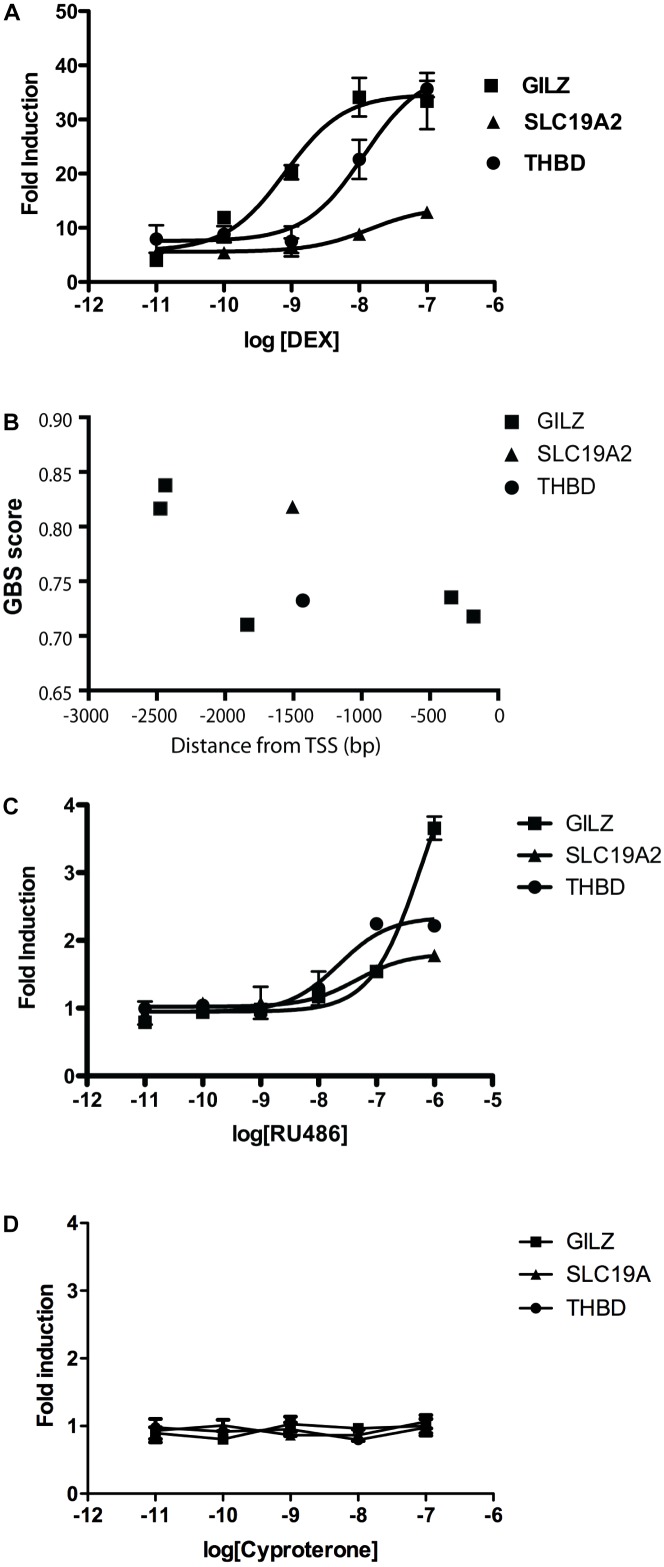
DEX and RU486, but not CYP, induce a dose-dependent and gene-specific response in A549 cells. **(A)** Gene expression response of three responsive genes, GILZ, SLC19A2, and THBD, to increasing concentrations of DEX measured by qRTPCR. Results are mean ± SEM of three independent experiments performed in triplicates. Fitted parameters are detailed in Table 1. **(B)** Binding affinity predictions corresponding to GREs present in proximal 5′UTR regions to transcription start sites (TSS) corresponding to GILZ, SLC19A2, and THBD. Results are expressed as GR binding scores (GBS), as previously described (Datson et al., 2011). **(C)** Gene expression response of three responsive genes, GILZ, SLC19A2, and THBD, to increasing concentrations of RU486 measured by qRTPCR. Results are mean ± SEM of three independent experiments performed in triplicates. Fitted parameters are detailed in Table 2. **(D)** Gene expression response of three responsive genes, GILZ, SLC19A2, and THBD, to increasing concentrations of CYP measured by qRTPCR. Results are mean ± SEM of three independent experiments performed in triplicates.

To model ligand-specific effects on GR-mediated gene transcription, we used three GR ligands with different pharmacological characteristics: DEX is a well-characterized GR agonist, while RU486 is usually described as a partial agonist (often used as antagonist), and CYP as a passive antagonist (Rousseau and Baxter, 1979; Honer et al., 2003; Meijer et al., 2005; Matthews et al., 2009). We studied the effects of increasing concentrations of DEX on the expression of three GR responsive genes in A549 cells. Glucocorticoid Induced Leucine Zipper (GILZ) is an important mediator of the anti-inflammatory effects of GCs (Ronchetti et al., 2015), THBD is an endothelial cell surface glycoprotein that controls thrombosis by downgrading thrombin-mediated fibrin generation and promoting protein C activation (Loghmani and Conway, 2018) and SLC19A2 is a thiamine transporter associated with the thiamine-responsive megaloblastic anemia syndrome (TRMA) (Aoyagi and Archer, 2011). In this cellular system, DEX dose-dependently induced the expression of the three genes tested, albeit with different pharmacological parameters. We found significant differences in calculated effective concentration 50 (EC_50_) and maximal response (*R*_max_) values. Specifically, DEX was significantly more potent in inducing GILZ than THBD or SLC19A2, and significantly less efficacious in inducing SLC19A2 than THBD or GILZ (Figure 1A and Table 1).

**Table 1:** Effect of DEX on gene expression in A549 cells.

Responsive gene	Parameter best fit value			
	pEC_50_	SEM	*R*_max_	SEM
GILZ	9.11	0.22	32.73	2.15
SLC19A2	7.85^∗^	0.30	13.86^∗^	1.50
THBD	7.90^∗^	0.18	37.06	4.26

*^∗^Significantly different from GILZ, p < 0.05, ANOVA.*

These differences are difficult to explain from the standpoint of theoretical models of ligand-induced gene expression because the ligand, the amount of receptors and all other components of the cellular environment were the same in all experiments. One possible explanation could be in the structure of the gene promoter and its GRE composition (Wang et al., 2004; So et al., 2008), suggesting that response magnitude depends on the strength of GR binding to GREs. However, numerous previous studies argue to the contrary (So et al., 2008; Pfaff and Fletterick, 2010; Dougherty et al., 2012; Chen et al., 2013), and previously published chromosome immunoprecipitation (ChIP) data analyzing these promoters do not predict all the differences in sensitivity to the GR agonist DEX (Wang et al., 2004). The promoter sequence data predicted GILZ to be strongly bound by the occupied GR with two high GR binding score (GBS) sites in its promoter, followed in order of predicted responsiveness by SLC19A, with one high GBS sites, and THBD with one low GBS site (Figure 1B), while DEX dose response curves showed a different order in both response efficacy (THBD∼GILZ>>SLC; Figure 1A) and potency (GILZ>>THBD∼SLC; Figure 1A). Similarly, RU486 induced significant changes in GILZ, SLC19A2 and THBD, expression in A549 cells, albeit only at high doses (Figure 1C and Table 2).

**Table 2:** Effect of RU486 on gene expression in A549 cells.

Responsive gene	Parameter best fit value			
	pEC_50_	SEM	*R*_max_	SEM
GILZ	6.21	0.16	5.32	0.66
SLC19A2	7.35^∗^	0.32	1.81^∗^	0.12
THBD	7.64^∗^	0.19	2.35^∗^	0.11

*^∗^Significantly different from GILZ, p < 0.05, ANOVA.*

**Table 3:** Effect of DEX, RU486, and CYP on GR translocation to the nucleus in A549 cells.

Ligand	Parameter best fit value			
	pEC_50_	SEM	*R*_max_	SEM
DEX	9.38	0.06	98.81	1.61
RU486	9.02	0.08	47.19^∗^	1.13
CYP	7.8^∗^	0.07	58.67^∗^	1.70

*^∗^Significantly different, p < 0.05, ANOVA.*

Importantly, the observed order of gene-transcription potency (SLC19A∼THBD>GILZ; Table 2) and efficacy (GILZ>SLC19A∼THBD; Table 2) induced by RU486 were different than those induced by DEX (Table 1). Noteworthy, although CYP alone did not have any detectable effect on the expression of the three genes analyzed (Figure 1D), DEX, RU486 and CYP induced GR translocation from the cytosol to the nucleus, indicating that the three ligands promote active changes on GR behavior and therefore cannot be ascribed as passive (or inactive) antagonists (Table 3).

To understand the differential, ligand- and gene-specific, biological behavior observed in A549 cells in more detail, we aimed to develop an MTC model that could integrate ligand–receptor–DNA–coregulator interactions.

### Development of a MTC Model for Ligand–Receptor–DNA–Coregulator Interactions

**FIGURE 2:**
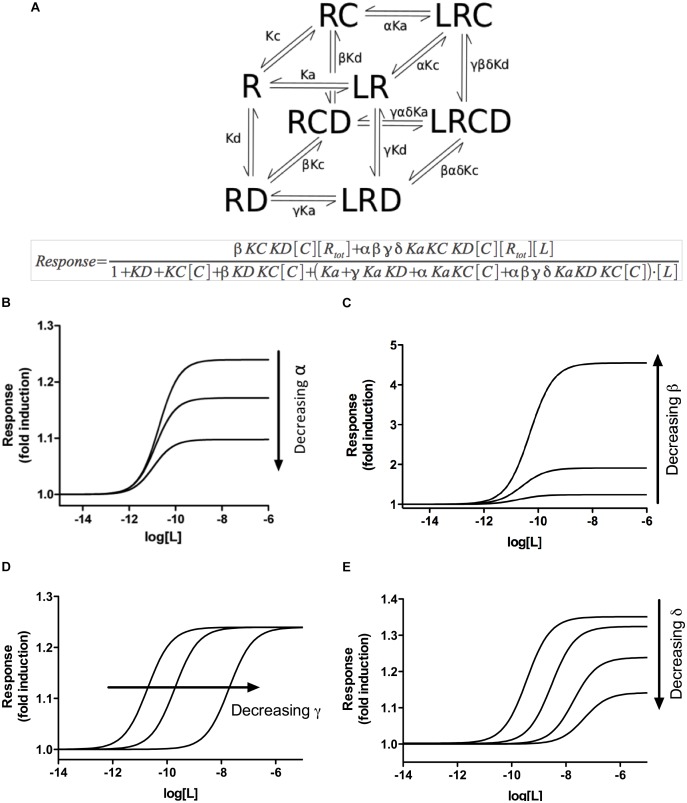
A multifactor complex model for ligand–nuclear receptor–DNA–coregulator interactions. **(A)** The simplest cubic representation of our model, based on the CTC model, describing the interactions between the GR (R), a ligand (L), a coregulator (C) and DNA (D). The equilibria indicated by arrows are governed by their corresponding equilibrium constants Ka, Kc, and Kd, which are modified by specific parameters α, β, γ, and δ described in Supplementary Information S1 and Supplementary Table S1. The mathematical depiction of the model is shown below **(A)**. **(B)** Simulation of the effect of variations in α values (representing the effect of ligand binding on the binding of the coregulator or vice versa) on ligand-dependent response, as indicated by the model. **(C)** Simulation of the effect of variations in β values (representing the effect of coregulator binding on the DNA binding, or vice versa) on ligand-dependent response, as indicated by the model. **(D)** Simulation of the effect of variations in γ values (representing the effect of ligand binding on the DNA binding, or vice versa) on ligand-dependent response, as indicated by the model. **(E)** Simulation of the effect of variations in δ values (representing how the binding of any two partners affects the binding of the third) on ligand-dependent response, as indicated by the model. Note that only when the δ parameter is taken into consideration, simultaneous variation in both EC_50_ and *R*_max_ can be simulated by the model.

An alternative hypothesis to explain the gene-specific response to DEX observed in A549 cells involves the differential recruitment of specific coregulators to the MTC active at each gene promoter, which could induce gene-specific expression. To model this situation, we postulated a theoretical equilibrium model of receptor action that explicitly includes the receptor (R), the ligand (L), the coregulator(s) (C), and the DNA (D) and four parameters that govern receptor species equilibria: α represents the effect of ligand binding on the binding of coregulator, β describes the effect of coregulator binding on the binding of receptor to DNA and γ the effect of ligand binding on the binding of receptor to DNA. In turn, δ represents the extent to which the joint effect of any two of ligand binding, coregulator binding or DNA binding varies conditional on the level of the third. A detailed description of all these parameters is presented as Supplementary Material and schematically in Figure 2. Our model assumes that the GR can spontaneously couple to the coregulators or the DNA even in the absence of the ligand (Power et al., 1991; Mani et al., 1994). Thus, the model is fully described by three basic equilibrium constants that account for ligand binding, coregulator coupling and DNA binding and four parameters that illustrate the interaction effect between them (Figure 2A and Supplementary Information S1).

**FIGURE 3:**
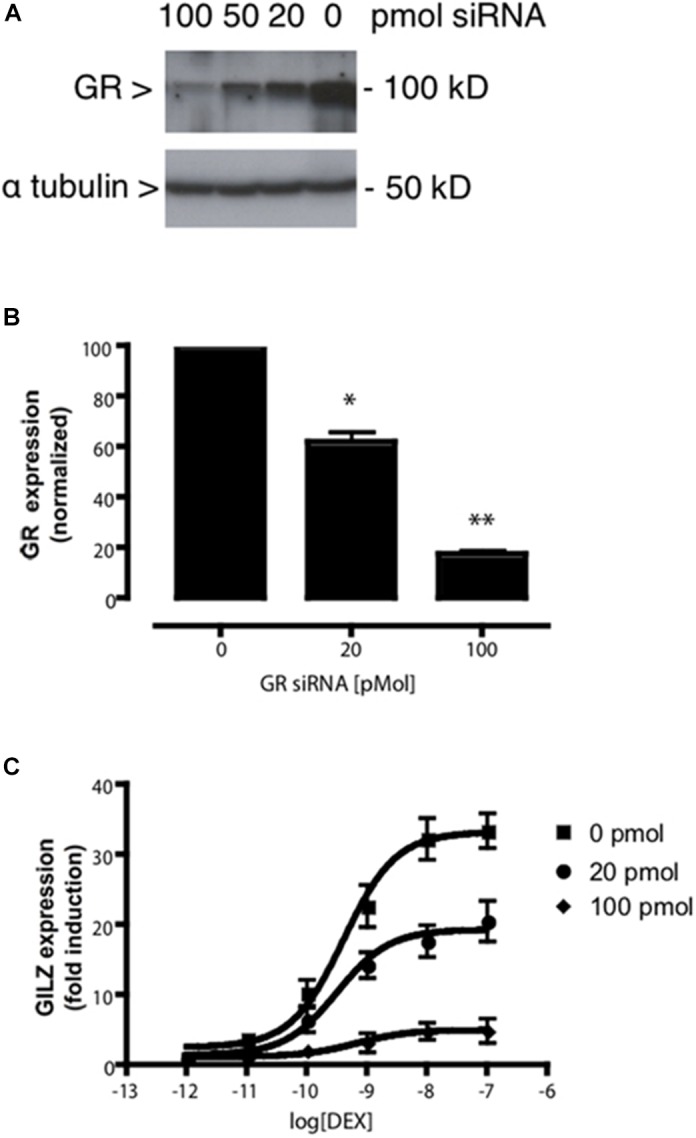
GR knockdown affects GILZ response to DEX. **(A)** Western blot depicting the effect of varying concentrations of a previously described specific siRNA targeting the GR (Fitzsimons et al., 2008) on GR protein levels in A549 cell lysates. The image shown is representative of five independent blots. **(B)** Quantification of the effect of varying concentrations of the specific siRNA used in **(A)** on GR expression. Results are expressed mean ± SEM of five independent blots. Statistically significant changes were identified using Student’s *t*-test. ^∗^*p* < 0.05; ^∗∗^*p* < 0.01. **(C)** DEX dose-dependent effect on GILZ expression at decreasing GR expression levels induced by siRNA-induced GR knockdown. The calculated EC_50_ values were not affected by GR knockdown, while GILZ maximal response to DEX was significantly attenuated by increasing GR knockdown (Table 4). DEX-induced GILZ expression was detectable even at maximal GR knockdown (100 pmol siRNA), indicating GILZ induction is robust even at low levels of GR expression.

Assuming that the MTC formed by the receptor, the ligand, the coregulator(s) and the DNA is responsible for the final induction of gene transcription, dose–response curves can be simulated with equations describing how the relative composition of the different components of the MTC affects ligand-dependent gene induction (Figure 2B–E). The model predicted a series of system characteristics that could be experimentally validated. According to the model, the concentration–response curve to a ligand can be modified adjusting the values assumed for each component of the MTC (Supplementary Information S1). Indeed, a first validation of the model using siRNAs to reduce GR expression in A459 cells resulted, as indicated by our model, in significant changes in *R*_max_ but not EC_50_ (Figure 3). Transfection of A549 cells with increasing siRNA concentrations resulted in concomitant decreases in GR expression (Figure 3A,B) and in GILZ’s *R*_max_ to DEX, without affecting its EC_50_ (Figure 3C and Table 4).

Importantly, when changes in the α, β, or γ parameters are simulated, the results predict changes in either *R*_max_ or EC_50_, but never both simultaneously (Figure 2B–D and Supplementary Information S1). This prediction leaves out the possibility that differences in coregulator recruitment or in DNA binding alone could explain the simultaneous change in EC_50_ and *R*_max_ observed in DEX concentration–response curves in A549 cells (Figure 1). However, when the δ parameter is taken into consideration, simultaneous variation in both EC_50_ and *R*_max_ can be explained theoretically using our model (Figure 2E). δ describes how two binding events impact on a third within the MTC proposed by the model, reflecting interaction between all the components of the complex. Therefore, our model suggests that non-independent binding events between the different components of the multifactor complex could be responsible for the simultaneous differences in EC_50_ and *R*_max_ to DEX observed in A549 cells (Figure 1A and Table 1).

The sequence of binding events leading to transcriptional activation by the GR has been intensively studied but not fully established yet (Rogatsky et al., 2003; John et al., 2009; Dougherty et al., 2012; Chen et al., 2013). It is considered to be a dynamic process and the most parsimonious hypothesis is that transcriptional activation by the GR, and NRs in general, involves multiple factors that act in both a sequential and combinatorial manner to reorganize chromatin templates (Pollard and Peterson, 1998; Glass and Rosenfeld, 2000; Voss et al., 2006; Stavreva et al., 2012). Moreover, the temporal order of the events leading to the formation and composition of the MTC that leads to transcription activation can take place in a gene- and cell-specific manner (Gronemeyer et al., 2004). Potential dissimilarities in the structure of promoters for GILZ, SLC19A, and THBD genes do not explain the differential expression patterns obtained with DEX and RU486 (Figure 1A vs. Figure 1C). This divergence can be explained by our MTC model, considering that each ligand induces a specific pattern of coregulator binding to GR forming the ternary complex LRC, which in turn may display differential affinity for DNA. Therefore, using a DNA-free system represented by the MARCoNI peptide array, we focused on understanding how the interactions between ligand and GR could cooperate to modulate coregulator binding.

### Ligand-Independent and Ligand–Specific Receptor–Coregulator Binding Events

Our model includes the existence of ligand-specific parameters governing multifactor complex formation (Zalachoras et al., 2013; Atucha et al., 2015) that can be tested experimentally in DNA-free conditions (Supplementary Information S1). Ligand affinity constant Ka (specific for each ligand), coregulator affinity constant Kc (specific for each individual coregulator NR-box), and the parameter α (characteristic of each ligand/coregulator pair) indicate how ligand and coregulator affects each other’s binding to the receptor and are key components of the model when only receptor, ligand, and coregulators are present. According to the model’s prediction, GR ligands should induce a characteristic coregulator binding profile as previously demonstrated (Zalachoras et al., 2013; Atucha et al., 2015).

**Table 4:** Effect of DEX on GILZ expression in A549 cells after siRNA-induced GR knockdown.

siRNA amount (pmol)	Parameter best fit value			
	pEC_50_	SEM	*R*_max_	SEM
0	9.37	0.15	33.18	1.57
20	9.29	0.22	19.28^∗^	1.27
100	9.17	0.54	4.97^∗^	0.78

*^∗^Significantly different, p < 0.05, ANOVA.*

**FIGURE 4:**
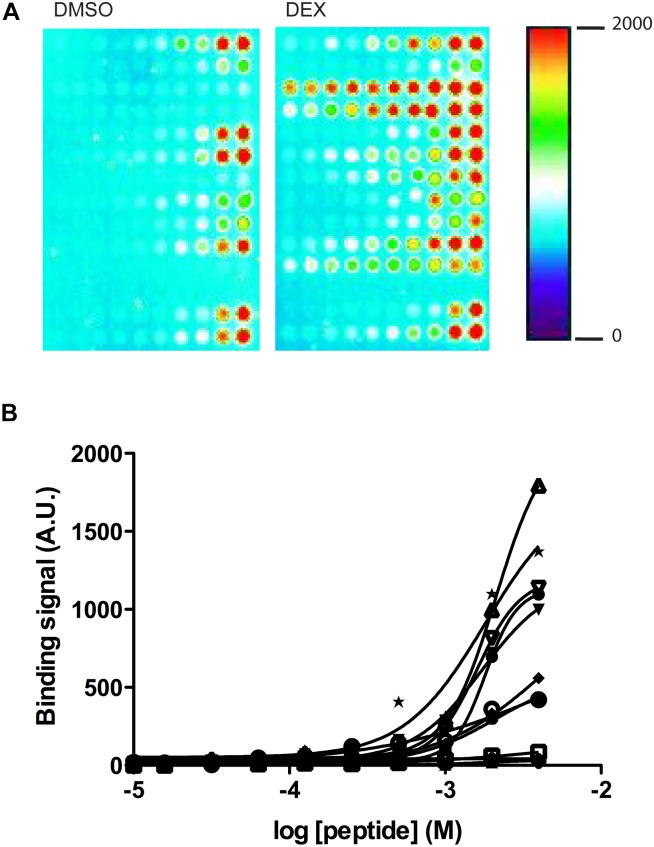
Quantification of GR LBD binding to coregulator-derived NR box peptides using a customized MARCoNI array. **(A)** Example image of the GR LBD binding signal detected from two MARCoNI arrays in the presence of vehicle (DMSO) or DEX. The heatmap indicates relative binding intensity. **(B)** Peptide dose-dependent binding isotherms detected for the 12 NR box peptides (Supplementary Table S3) in the absence of any GR ligand (vehicle = DMSO). (•) NRIP1_LxxLL185_173_195, (x) NRIP1_LxxLL21_8_30, (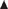) NRIP1_LxxLL266_253_275_C263S, (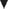) NRIP1_LxxLL380_368_390, (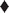) NRIP1_LxxLL500_488_510, (ο) NRIP1_LxxLL713_700_722, (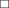) NRIP1_LxxLL819_805_831, (Δ) NRIP1_LxxLL936_924_946, (∇) NRIP1_LxxML1068_1055_1077, (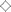) PRGC1_LxxLL144_130_155, (•) PPRB_LxxLL645_632_655, (∗) ZNHI3_LxxLL101_89_111. Results are expressed mean ± SEM of three independent experiments. The molar annotation as concentration refers to the molar concentration of the peptides in the spot solution.

**FIGURE 5:**
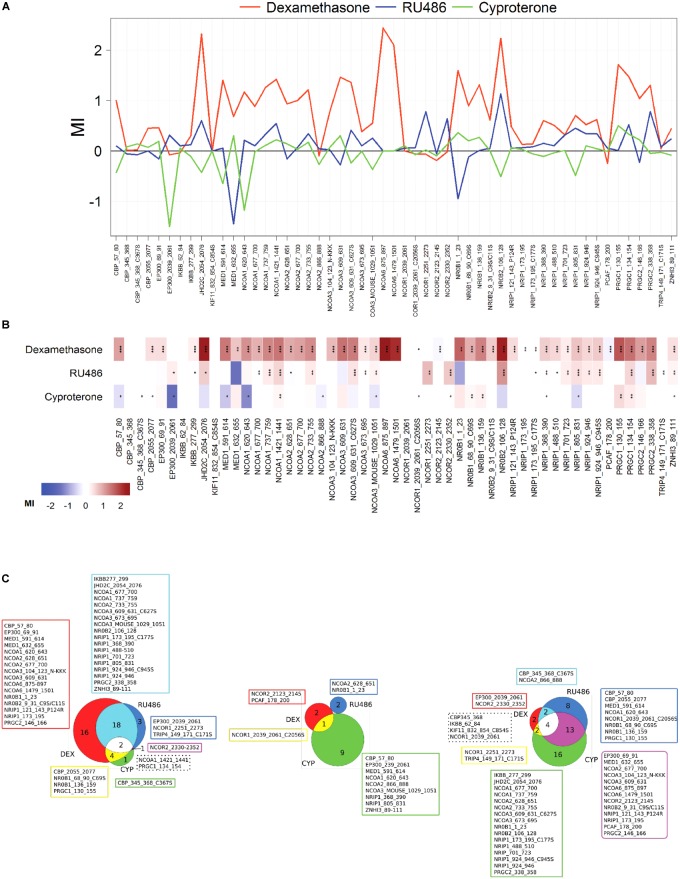
DEX, RU486 and CYP induce specific GR LBD-to-NR box peptide binding profiles. **(A)** Characteristic binding profiles induced by 1 × 10^-7^ DEX (red), RU486 (blue) or CYP (green) obtained using the quantitative *in vitro* assay, MARCoNI. Modulation Index (MI) > 0 suggests ligand-favored binding, while MI < 0 suggests ligand-disfavored binding of a peptide compared to DMSO. **(B)** Heatmap depiction of details of ligand-induced binding of coregulator peptides using MARCoNI. **(C)** Venn diagrams showing the number of peptides whose binding was favored (left), unfavored (center) or unchanged (right) by GR ligands. In all cases, statistically significant changes relative to DMSO were identified by Student’s *t*-test. ^∗^*p* < 0.05, ^∗∗^*p* < 0.01 or ^∗∗∗^*p* < 0.001.

Ligand-independent and -dependent coregulator binding profiles were measured using a MARCoNI peptide array. This array contained 53 coregulator-derived peptides representing a wide range of coregulator NR-boxes known to interact with the GR and other nuclear receptors, which sequence details, Gene Name and UniProt Knowledge Base accession numbers are shown in Supplementary Table S2. First, NR box peptides were titrated against the GR LBD in the absence of ligand using a customized MARCoNI array (Figure 4). A concentration series of each NR box peptide was immobilized on the array and incubated with 3 nM of the GR LBD and binding isotherms were calculated from these data. We observed a significant basal binding of the GR LBD to several NR-box peptides in the absence of any ligand (Figure 4A). This basal coregulator recruitment in the absence of ligand is predicted by the model (RC species in Figure 2) and is in agreement with similar observations done using fluorescence polarization assays (Pfaff and Fletterick, 2010). Twelve NR box peptides showed positive ligand-independent binding to the GR LBD, although with variable binding profiles (Supplementary Table S3). Ten of these NR box peptides showed lower affinity binding profiles, while two NR box peptides showed higher affinity binding. These higher affinity binding peptides corresponded to the coactivator PRGC1 and the corepressor NRIP1 (Figure 4B and Supplementary Table S3). Secondly, we used a MARCoNI array in which 1 mM of each NR box peptide was immobilized and incubated with 3 nM of the apo GR LBD in the presence of a receptor-saturating concentration of three selected GR ligands or a vehicle control, to study the effect of GR ligands on the basal binding of GR LBD to NR box peptides. We analyzed the coregulator binding profile induced by DEX, RU486, and CYP. These three ligands induced characteristic coregulator binding profiles, in some cases favoring and, in some others, disfavoring the basal binding of the apo GR LBD to NR box peptides observed in the absence of ligand (Figure 5). Notably, the NR box peptide binding profile induced by RU486 resembled for some peptides the effect induced by DEX, although RU486’s effects were substantially weaker in many cases, reflecting its partial agonist activity (Figure 5A,B). Interestingly, GR LBD binding to NR box peptides from the coactivators NCOA2, NCOA3 and NR0B1 was strongly favored by DEX, while it was disfavored by RU486 (Figure 5A,B), suggesting that this differential effect on coregulator binding is involved in the differential pharmacological effects of the two ligands. Supporting this preliminary conclusion, we did not find any example of the opposite pattern in our dataset, this is, binding to NR box peptides that were disfavored by DEX but promoted by RU486.

In contrast, the NR box peptide binding profile induced by CYP was more divergent from that induced by DEX (Figure 5B,C). We did not find any example in our dataset of NR box peptides for which binding to GR was favored by CYP but disfavored by DEX (Figure 5B). Interestingly, from the 10 NR box peptides whose binding was disfavored by CYP, 7 were favored by DEX (Figure 5B), suggesting that these differences in coregulator binding may be crucial to understand the pharmacological differences between DEX and CYP.

**FIGURE 6:**
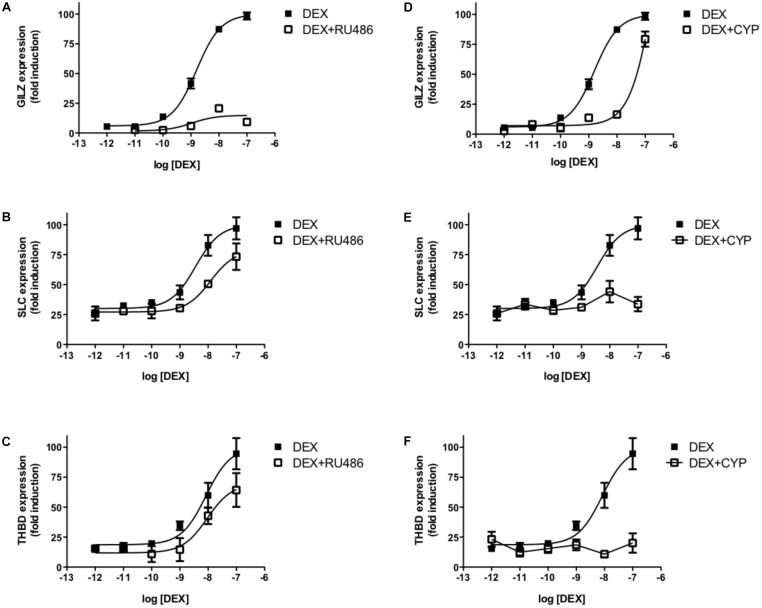
RU486 and CYP differentially affect DEX-induced gene expression. Transcriptional response of three GR-responsive genes, GILZ, SLC19A2, and THBD, to increasing concentrations of DEX measured by qRTPCR. Cells were preincubated with 10^-7^M RU486 **(A,C,E)** or CYP **(B,D,F)**. Results are expressed as mean ± SEM of three independent experiments performed in triplicates.

In summary, the pharmacological behaviors observed for the three GR ligands used in experiments in A549 cells (Figure 1) can be interpreted using our MTC model considering the interactions between receptor–ligand–coregulator–DNA binding events. Our model suggests that there exist an allosteric phenomenon involving the joint effect of the three GR partners within the MTC (L; C and D), reflected by the δ factor, i.e., a specific ligand induces the recruitment of a specific set of coregulators, that differentially affects the expression of a particular gene. In fact, our experimental observations *in vitro* with the MARCoNI, support the induction of ligand-specific binding profiles between the GR LBD and NR box peptides (Zalachoras et al., 2013; Atucha et al., 2015) that can explain the differential gene-specific behaviors of GC ligands. The model indicates that the distinctive ligand-specific binding of GR to coregulators would differentially impact on the expression of specific genes when cells are co-incubated with DEX and RU486 or CYP. Indeed, Figure 6 shows that RU486 can block DEX-induced GILZ expression without affecting SLC and THBD expression significantly, while CYP specifically blocked DEX-induced SLC and THBD expression without affecting GILZ maximal expression levels (Figure 6).

**FIGURE 7:**
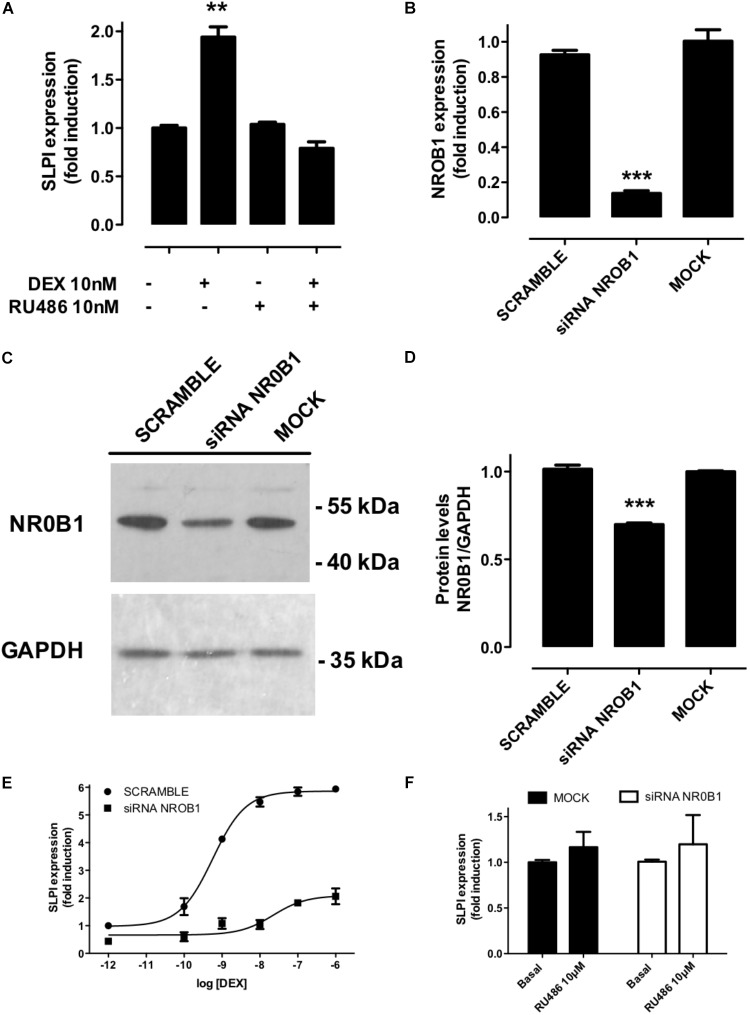
NR0B1 knockdown affects SLPI response to DEX but not to RU486. **(A)** DEX but not RU486 induces SLPI expression in A549 cells, as expected, coincubation with RU486 blocked DEX-mediated effects, ^∗∗^*p* < 0.01. **(B)** Effect of cell transfection with siRNA targeting NR0B1 on mRNA and **(C,D)** protein levels in A549 cells measured by qRTPCR and Western-blot, respectively. The expression of the glyceraldehyde 3-phosphate dehydrogenase (GADPH) was used as internal control and normalization in **(C,D)**, ^∗∗∗^*p* < 0.001. **(E)** Transcriptional response of SLPI to increasing concentrations of DEX measured by qRTPCR in cell transfected with specific siRNAs against NR0B1 or a scramble siRNA control. The fitted parameters are detailed in Table 5. **(F)** Transcriptional response of SLPI to RU486 measured by qRTPCR. Results are expressed as mean ± SEM of three independent experiments performed in triplicates.

According to the MTC, each ligand may induce a specific response by recruiting a defined array of cofactors. Among the coregulators that were differentially bound by GR ligands, we chose NR0B1 as a proof-of-concept experimental validation. NR0B1 displayed a maximum difference between DEX-favored and RU486-disfavored binding to the GR LBD *in vitro* (Figure 5). SLPI, a protease inhibitor expressed by cells at mucosal surfaces, is stimulated by IL-1β. IL-1 β-induced SLPI expression is increased by DEX and inhibited by RU486 in A549 cells (Ito et al., 2001). We found that SLPI expression is strongly induced by DEX but not by RU486 in non-transfected A549 cells (Figure 7A). While NR0B1 downregulation using specific siRNAs (Figure 7B–D) significantly diminished the maximal DEX-induced expression of SLPI (Figure 7E) and simultaneously increased DEX’s EC_50_ for SLPI induction, it did not affect RU486-induced SLPI expression (Figure 7F), demonstrating its specific role in DEX-induced SLPI expression in A549 cells.

## Discussion

In this work, we have developed a mathematical model describing the interactions between the GR and other components of an MTC that control the transcription of GR-target genes. This model is based on basic equilibrium equations governed by three constants and four parameters accounting for ligand, coregulator and DNA binding events and accommodates plausible non-independent effects among them. Here, we show proof-of concept for the model using A459 cells, a well characterized system to study GR-mediated activation of responsive genes (So et al., 2007), and three extensively characterized GR ligands; DEX, RU486, and CYP. Future research should be aimed to extensively validate the model using multiple cell lines, ligands, coregulators, and responsive genes.

Factors that regulate gene transcription are assembled in multicomponent complexes by combinatorial interactions (Britten and Davidson, 1969; Gierer, 1974; Yamamoto et al., 1998). In this context, the GR provides a well-characterized and physiologically relevant study system in which the effects of ligand dose and chemistry, treatment duration and kinetics, interaction with coregulatory factors and DNA binding site sequence and structure have all been intensively studied (Darimont et al., 1998; Collingwood et al., 1999; Rogatsky et al., 2003; John et al., 2009; Reddy et al., 2009; Stavreva et al., 2009). Interestingly, many of these factors induce allosteric changes on the GR (Meijsing et al., 2009; Pfaff and Fletterick, 2010; Wang et al., 2012; Watson et al., 2013), suggesting that allosteric conformational changes induced by components of the MTC are crucial to understanding GR-mediated gene transcription. In fact, a model based on such a “conformational ensemble” has been proposed to explain ligand-induced switch between ER-α- mediated genomic and non-genomic effects (Norman et al., 2004).

One of the significant challenges in modeling GR-mediated gene transcription is to understand how a multiple step reaction sequence can result in dose response curves that follow a Michaelis–Menten function as we show here in A549 cells. Explaining this experimental observation requires careful consideration of parameters such as EC_50_ and *R*_max_ (Simons and Chow, 2012). Consequently, a theoretical framework able to predict changes in shape, position and *R*_max_ of ligand dose-dependent response has been developed (Ong et al., 2010). This model suggests that full dose–response curves need to be considered to achieve complete mechanistic insight into GR-mediated gene transcription. The model we describe here is in agreement with this concept and is conceptually based on the Cubic Ternary Complex Receptor-Occupancy Model (CTC) originally proposed by Weiss et al. (1996). The CTC has been successfully used to describe interactions between receptors and their interacting partners, and to predict dose–response curves for many ligand/receptor pairs (Weiss et al., 1996). Indeed, due to its theoretical completeness, the application of the CTC framework has allowed us to experimentally characterize low-abundance receptor states predicted by the model in other systems (Monczor et al., 2003; Fitzsimons et al., 2004; Tubio et al., 2010). Following the law of mass action, our model describes the interactions between a ligand, a receptor and accessory molecules, including coregulators and DNA sequences resulting in eight receptor species that coexist at equilibrium; the free receptor (R) and receptor species bound to the ligand (LR), to the accessory coregulators or DNA (RC and RD, respectively), and bound to any two or three factors (LRC, LRD, RCD, LRCD) (Figure 2 and Supplementary Information S1). There are several mechanisms by which GR modulates gene expression. We decided to experimentally validate our model measuring GR mediated gene transactivation because the model’s assumptions reflect better transactivation mechanisms, since GC-induced gene transactivation is mediated by the direct interaction of GR with DNA and cofactors, while gene repression is largely based on by protein:protein interactions (Ratman et al., 2013).

**Table 5:** Effect of DEX on SLPI expression in A549 cells after siRNA-induced NR0B1 knockdown.

Condition	Parameter best fit value			
	pEC_50_	SEM	*R*_max_	SEM
SCRAMBLE	9.25	0.05	5.86	0.07
siRNA NR0B1	7.65^∗^	0.28	2.09^∗^	0.15

*^∗^Significantly different from SCRAMBLE, p < 0.05, ANOVA.*

The experimental validation of predictions of this model in A549 cells, a well-characterized system to study GR-dependent gene expression (So et al., 2007), and *in vitro* using a system of real time quantification of GR-coregulator interactions, suggests that the model can help to interpret specific ligand dose-dependent effects on gene expression, and could be used to explain the pharmacological characteristics (e.g., the simultaneous variation in relative potency and efficacy) of ligand-specific and gene-specific transcriptional regulation by the GR that are difficult to explain using alternative models based only on ligand potency. One of the distinctive features of our model is that receptors may spontaneously bind any partner without binding the ligand. This feature is particularly interesting for the exploration of pharmacological properties of GR ligands that seem to behave as neutral antagonists *in vivo* and as partial agonists *in vitro* such as RU486 and the non-steroidal CP-472555 (Weigel and Zhang, 1998). We verified the gene-specific behavior indicated by our model for RU486 and DEX in A549 cells. Interestingly, SLC19A2 and THBD showed 50% gene expression response (EC_50_) values for DEX in the low nM range (Table 1), in agreement with previous reports (Reddy et al., 2009). However, GILZ robustly responded to significantly lower concentrations of DEX (Table 1). At least one other gene, PER1, with similar high sensitivity to DEX has been shown to have similar characteristics and its increased responsiveness could not be explained by GRE composition or Pol II occupancy in its promoters (Reddy et al., 2009), suggesting that differential binding to coregulators maybe involved. The order of gene induction potency and efficacy observed for the partial agonist RU486 was different from the one observed for the full agonist DEX, a crucial observation that cannot be explained using conventional pharmacological models based only on ligand potency. We have previously explored the possible physiological relevance of downregulating the GR using RNA interference in the mouse brain, showing that this experimental approach is technically feasible *in vivo* (Fitzsimons et al., 2013). The results presented here using siRNAs to downregulate GR expression in A549 cells indicate that GR downregulation may result in a decrease of the maximal response to GR without affecting its EC_50_, which we exemplify using DEX and one GR responsive gene, GILZ. If these results could be generalized they may be relevant to further understand the physiopathological relevance of the expression of dominant-negative GR isoforms, such GR-beta (Yudt et al., 2003; Lewis-Tuffin and Cidlowski, 2006), which has been associated with decreased GC responsiveness and susceptibility to develop autoimmune diseases (Tait et al., 2008).

Using the *in vitro* system provided by the MARCoNI chip, we observe active binding of the GR-LBD to both coactivators and corepressors in the absence of ligand. This observation is compatible with similar ones made before using the GR-LBD (Pfaff and Fletterick, 2010). Possibly, full-length GR species that spontaneously bind full-length coregulators may exist at very low levels, perhaps undetectable or destabilized in most cell-based assays and thus, their existence *in vivo* will require further experimental validation. Furthermore, the GR amino terminal end, not included in the recombinant peptide used in our MARCoNI assay, also contains binding sites for several coregulators (Godowski et al., 1987; Eickelberg et al., 1999; Siriani et al., 2003). Importantly, the notion that the GR may exist in native conformation ensembles capable to assuming active and inactive behaviors is compatible with the conformation ensemble model proposed to explain cellular behaviors mediated by the ER-alpha (Norman et al., 2004). Several studies suggest that the GR is refractory to ligand-independent activation (reviewed in Weigel and Zhang, 1998). Others have suggested that the GR can be activated in the absence of hormone (Cenni and Picard, 1999; Eickelberg et al., 1999). Mutagenesis studies demonstrated that single amino acid mutations and phosphorylation events can render the GR constitutively active in the absence of hormone (Godowski et al., 1987), reinforcing the idea that GR conformational states allowing ligand-independent activity exist, albeit in low abundance with respect to the inactive forms. Supporting this hypothesis, some studies have demonstrated the ability of the GR to regulate gene expression through non-hormone-binding forms of the receptor in overexpression systems (Siriani et al., 2003). Therefore, the CTC framework provides a theoretical environment to understand and interpret GR behaviors that may be mediated by low abundance receptor states (Tubio et al., 2010).

Here, we introduce the application of the most parsimonious version of the CTC to understand GR-mediated cellular behaviors. The model (Figure 2) includes one GR, one ligand, one coregulator and the DNA. This is a representation including all the thermodynamic equilibria between the four partners involved in the MTC and results in a convenient cubic depiction of the eight receptor species discussed above. This representation cannot accommodate simultaneous interactions with more than one coregulator, which could be expected given the well-characterized complexity of the MTC (McKenna and O’Malley, 2002). However, the basic cubic structure of the CTC can be easily extended to include more than one accessory species, such as multiple coregulators. This is done by simply joining two cubes, differing only in their accessory species, by their accessory species-free face resulting in a model represented by a double cube. If more than one accessory species is to be added, the model cannot be visualized in three dimensions but can still be modeled and analyzed mathematically (Weiss et al., 1996). Similarly, the GR is the product of a single gene from which multiple transcriptional and translational isoforms are generated through alternative splicing and alternative translation initiation. Multiple studies have shown before that the specificity of GR signaling may arise, at least in part, from this molecular diversity, because some of these isoforms have differential ligand affinities and efficacy to induce the expression of target genes (Cain and Cidlowski, 2015). This complexity seems difficult to grasp, however, in terms of our model each GR isoform could be modeled using a different CTC with specific affinity constants as described in Figure 2. Although admittedly practically cumbersome, this procedure does not impose theoretical limitations for our model. With respect to the validity of the model predictions presented in the manuscript, our *in vitro* experiments were done with the LBD of the human GR-alpha, which is the most abundant splice variant of the GR in most tissues (Pujols et al., 2002). For the cellular studies that we performed in A549 cells, the full-length GR mRNA expressed there may present N-terminal translation variants (Lu and Cidlowski, 2005), and these go undefined in the vast majority of studies. However, in our case this diversity in N-terminal translation variants may be irrelevant, as the coregulator interactions that we model concern the LBD (AF-2). Therefore, by including all possible thermodynamic equilibria between the species involved, our model considers the non-independent effect of multiple factor binding events on GR ligand dose–response curves.

An important prediction of our model is that a basal GR-coregulator binding profile is differentially modulated in a ligand-specific manner. All the receptor species present in a ligand-free environment, i.e., R, RC, RCD and RD are induced to form LR, LRC, LRCD and LRD in the presence of a ligand and this induction is governed by the ligand-specific constant and parameters Ka and α, γ and δ (Figure 2). Our observations support the view that the changes induced by different ligands on a basal profile of GR binding to coregulators could play a significant role in cell type and tissue-specific ligand actions, such as those observed before with RU486 and other selective SGRMs (Zalachoras et al., 2013; Atucha et al., 2015). We performed a proof-of-concept validation by downregulation NR0B1 in A549 cells (Figure 7). Downregulation of NR0B1 levels resulted in a significant decrease in DEX-induced SLPI expression, without affecting SLPI’s lack of response to RU486. These data indicate that gene-specific response to GR ligands can be, at least partially, regulated by coregulator abundance.

Our model predicts a number of complex interactions between different molecular species engaged in the MTC, however, we have not been able to validate all of them experimentally. Importantly, all the interactions in the MTC may be changed upon posttranslational modifications of the GR or the coregulator proteins, that may affect the affinity of the protein:protein interactions or the localization of the proteins involved. The interactions between DNA and other components of the MTC are particularly challenging because their full understanding would require characterization in living cells, where these interactions are most relevant. DNA has been proposed to act as an allosteric ligand of the GR in cell-based assays (Pfaff and Fletterick, 2010), we face technical limitations of the MARCoNI array to experimentally measure interactions with DNA. In view of these limitations, in the present work we focused on interactions between the GR and coregulators. There are other factors, such as variations in DNA binding motives, number of GREs, the role of chromatin structure and epigenetic factors, which may influence DNA binding of the GR or other components of the MTC. In our model, these are variations on the theme of specific sequence and structure of individual GREs – the parameters in the cubic model will depend on specific sequence- be it genomically or epigenetically determined. Thus, these variations are automatically incorporated – per GRE-dependent process, in the model. Our experimental validation incorporates cell line-specific effects of the GR assuming that they will, at least in part, result from differences in coregulator expression. In this respect, we used A549 cells in our primary validation of the GR-responsive genes used in this work because these were previously extensively characterized in this cell line, including their GRE composition and sequence, thus leaving out factors that would only introduce uncertainty in our validation steps such as variations in DNA binding motives, number of GREs (Wang et al., 2004) and epigenetic factors (Reul et al., 2009), mentioned before. In conclusion, our theoretical model based on the mathematical framework of the CTC is able to accurately interpret a variety of GR behaviors in different experimental setups, ranging from interaction with coregulator binding *in vitro* to differential effects on gene expression in A549 cells and may be used to characterize and interpret ligand- and gene-specific effects on transcriptional activity. Our observations may explain previous reports of RU486 and CYP having (partial) agonistic activities and justifies their classification as SGRMs, in the sense that they interact differentially with subsets of the GR functions induced by the full agonist (Gronemeyer et al., 2004), probably in a gene-specific manner as indicated by our model and the coregulator binding profiles they induce *in vitro*.

## Data Availability

All datasets generated for this study are included in the manuscript and/or the Supplementary Files.

## Author Contributions

CF and FM developed the model, designed the study, and wrote the manuscript. AC, CZ, and RH performed the experiments. OM supervised the experiments and corrected the manuscript. All authors analyzed and interpreted the data and revised the manuscript critically for important intellectual content and approved the final version.

## Conflict of Interest Statement

RH is employed by PamGene International B.V. The remaining authors declare that the research was conducted in the absence of any commercial or financial relationships that could be construed as a potential conflict of interest.

## Footnotes

1 http://primer3.ut.ee/

2 http://www.ncbi.nlm.nih.gov/BLAST

3 http://imagej.nih.gov/ij/

4 http://www.cellprofiler.org/

## Abbreviations

CYP: cyproterone
DEX: dexamethasone
GC: glucocorticoid
GR: glucocorticoid receptor
GRE: glucocorticoid responsive element
MARCoNI: Microarray Assay for Real-time Coregulator-Nuclear Receptor Interaction
MTC: multifactorial transcriptional complex

## References

[B1] Adams M. Meijer O. C. Wang J. Bhargava A. Pearce D. (2003). Homodimerization of the glucocorticoid receptor is not essential for response element binding: activation of the phenylethanolamine N-methyltransferase gene by dimerization-defective mutants. *Mol. Endocrinol. Baltim.* 17 2583–2592. 10.1210/me.2002-0305 12933902

[B2] Aoyagi S. Archer T. K. (2011). Differential glucocorticoid receptor-mediated transcription mechanisms. *J. Biol. Chem.* 286 4610–4619. 10.1074/jbc.M110.195040 21127044 PMC3039389

[B3] Atucha E. Zalachoras I. van den Heuvel J. K. van Weert L. T. Melchers D. Mol I. M. (2015). A mixed glucocorticoid/mineralocorticoid selective modulator with dominant antagonism in the male rat brain. *Endocrinology* 156 4105–4114. 10.1210/en.2015-1390 26305887

[B4] Bain D. L. Connaghan K. D. Maluf N. K. Yang Q. Miura M. T. De Angelis R. W. (2014). Steroid receptor-DNA interactions: toward a quantitative connection between energetics and transcriptional regulation. *Nucleic Acids Res.* 42 691–700. 10.1093/nar/gkt859 24064251 PMC3902896

[B5] Bolton E. C. So A. Y. Chaivorapol C. Haqq C. M. Li H. Yamamoto K. R. (2007). Cell- and gene-specific regulation of primary target genes by the androgen receptor. *Genes Dev.* 21 2005–2017. 10.1101/gad.1564207 17699749 PMC1948856

[B6] Britten R. J. Davidson E. H. (1969). Gene regulation for higher cells: a theory. *Science* 165 349–357. 10.1126/science.165.3891.349 5789433

[B7] Cain D. W. Cidlowski J. A. (2015). Specificity and sensitivity of glucocorticoid signaling in health and disease. *Best Pract. Res. Clin. Endocrinol. Metab.* 29 545–556. 10.1016/j.beem.2015.04.007 26303082 PMC4549805

[B8] Carpenter B. McKay M. Dundas S. R. Lawrie L. C. Telfer C. Murray G. I. (2006). Heterogeneous nuclear ribonucleoprotein K is over expressed, aberrantly localised and is associated with poor prognosis in colorectal cancer. *Br. J. Cancer* 95 921–927. 10.1038/sj.bjc.6603349 16953238 PMC2360539

[B9] Cenni B. Picard D. (1999). Ligand-independent activation of steroid receptors: new roles for old players. *Trends Endocrinol. Metab.* 10 41–46. 10.1016/S1043-2760(98)00121-0 10322393

[B10] Chen S.-H. Masuno K. Cooper S. B. Yamamoto K. R. (2013). Incoherent feed-forward regulatory logic underpinning glucocorticoid receptor action. *Proc. Natl. Acad. Sci. U. S. A.* 110 1964–1969. 10.1073/pnas.1216108110 23307810 PMC3562826

[B11] Chow C. C. Ong K. M. Dougherty E. J. Simons S. S. (2011). Inferring mechanisms from dose-response curves. *Methods Enzymol.* 487 465–483. 10.1016/B978-0-12-381270-4.00016-0 21187235 PMC3177954

[B12] Coghlan M. J. Jacobson P. B. Lane B. Nakane M. Lin C. W. Elmore S. W. (2003). A novel antiinflammatory maintains glucocorticoid efficacy with reduced side effects. *Mol. Endocrinol.* 17 860–869. 10.1210/me.2002-0355 12586843

[B13] Collingwood T. N. Urnov F. D. Wolffe A. P. (1999). Nuclear receptors: coactivators, corepressors and chromatin remodeling in the control of transcription. *J. Mol. Endocrinol.* 23 255–275. 10.1677/jme.0.0230255 10601972

[B14] Darimont B. D. Wagner R. L. Apriletti J. W. Stallcup M. R. Kushner P. J. Baxter J. D. (1998). Structure and specificity of nuclear receptor-coactivator interactions. *Genes Dev.* 12 3343–3356. 10.1101/gad.12.21.3343 9808622 PMC317236

[B15] Datson N. A. Polman J. A. de Jonge R. T. van Boheemen P. T. M. van Maanen E. M. T. Welten J. (2011). Specific regulatory motifs predict glucocorticoid responsiveness of hippocampal gene expression. *Endocrinology* 152 3749–3757. 10.1210/en.2011-0287 21846803

[B16] Dougherty E. J. Guo C. Simons S. S. Chow C. C. (2012). Deducing the temporal order of cofactor function in ligand-regulated gene transcription: theory and experimental verification. *PloS One* 7:e30225. 10.1371/journal.pone.0030225 22272313 PMC3260260

[B17] Eickelberg O. Roth M. Lörx R. Bruce V. Rüdiger J. Johnson M. (1999). Ligand-independent activation of the glucocorticoid receptor by beta2-adrenergic receptor agonists in primary human lung fibroblasts and vascular smooth muscle cells. *J. Biol. Chem.* 274 1005–1010. 10.1074/jbc.274.2.1005 9873044

[B18] Estébanez-Perpiñá E. Moore J. M. R. Mar E. Delgado-Rodrigues E. Nguyen P. Baxter J. D. (2005). The molecular mechanisms of coactivator utilization in ligand-dependent transactivation by the androgen receptor. *J. Biol. Chem.* 280 8060–8068. 10.1074/jbc.M407046200 15563469

[B19] Fitzsimons C. P. Ahmed S. Wittevrongel C. F. W. Schouten T. G. Dijkmans T. F. Scheenen W. J. (2008). The microtubule-associated protein doublecortin-like regulates the transport of the glucocorticoid receptor in neuronal progenitor cells. *Mol. Endocrinol.* 22 248–262. 10.1210/me.2007-0233 17975023 PMC5419639

[B20] Fitzsimons C. P. Monczor F. Fernández N. Shayo C. Davio C. (2004). Mepyramine, a histamine H1 receptor inverse agonist, binds preferentially to a G protein-coupled form of the receptor and sequesters G protein. *J. Biol. Chem.* 279 34431–34439. 10.1074/jbc.M400738200 15192105

[B21] Fitzsimons C. P. van Hooijdonk L. W. A. Schouten M. Zalachoras I. Brinks V. Zheng T. (2013). Knockdown of the glucocorticoid receptor alters functional integration of newborn neurons in the adult hippocampus and impairs fear-motivated behavior. *Mol. Psychiatry* 18 993–1005. 10.1038/mp.2012.123 22925833

[B22] Gertz J. Savic D. Varley K. E. Partridge E. C. Safi A. Jain P. (2013). Distinct properties of cell-type-specific and shared transcription factor binding sites. *Mol. Cell* 52 25–36. 10.1016/j.molcel.2013.08.037 24076218 PMC3811135

[B23] Gierer A. (1974). Molecular models and combinatorial principles in cell differentiation and morphogenesis. *Cold Spring Harb. Symp. Quant. Biol.* 38 951–961. 10.1101/SQB.1974.038.01.097 4524796

[B24] Glass C. K. Rosenfeld M. G. (2000). The coregulator exchange in transcriptional functions of nuclear receptors. *Genes Dev.* 14 121–141. 10652267

[B25] Godowski P. J. Rusconi S. Miesfeld R. Yamamoto K. R. (1987). Glucocorticoid receptor mutants that are constitutive activators of transcriptional enhancement. *Nature* 325 365–368. 10.1038/325365a0 3808033

[B26] Granja-Galeano G. Zappia C. D. Fabián L. Davio C. Shayo C. Fernández N. (2017). Effect of mutation of Phe 2436.*44* of the histamine H2 receptor on cimetidine and ranitidine mechanism of action. *Biochem. Pharmacol.* 146 117–126. 10.1016/j.bcp.2017.09.014 28962836

[B27] Gronemeyer H. Gustafsson J.-A. Laudet V. (2004). Principles for modulation of the nuclear receptor superfamily. *Nat. Rev. Drug Discov.* 3 950–964. 10.1038/nrd1551 15520817

[B28] Heery D. M. Kalkhoven E. Hoare S. Parker M. G. (1997). A signature motif in transcriptional co-activators mediates binding to nuclear receptors. *Nature* 387 733–736. 10.1038/42750 9192902

[B29] Honer C. Nam K. Fink C. Marshall P. Ksander G. Chatelain R. E. (2003). Glucocorticoid receptor antagonism by cyproterone acetate and RU486. *Mol. Pharmacol.* 63 1012–1020. 10.1124/mol.63.5.1012 12695529

[B30] Hur E. Pfaff S. J. Payne E. S. Grøn H. Buehrer B. M. Fletterick R. J. (2004). Recognition and accommodation at the androgen receptor coactivator binding interface. *PLoS Biol.* 2:E274. 10.1371/journal.pbio.0020274 15328534 PMC509409

[B31] Ito K. Jazrawi E. Cosio B. Barnes P. J. Adcock I. M. (2001). p65-activated histone acetyltransferase activity is repressed by glucocorticoids: mifepristone fails to recruit hdac2 to the p65-hat complex. *J. Biol. Chem.* 276 30208–30215. 10.1074/jbc.M103604200 11395507

[B32] John S. Johnson T. A. Sung M.-H. Biddie S. C. Trump S. Koch-Paiz C. A. (2009). Kinetic complexity of the global response to glucocorticoid receptor action. *Endocrinology* 150 1766–1774. 10.1210/en.2008-0863 19131569 PMC2659280

[B33] Kenakin T. (2004). Principles: receptor theory in pharmacology. *Trends Pharmacol. Sci.* 25 186–192. 10.1016/j.tips.2004.02.012 15063082

[B34] Koppen A. Houtman R. Pijnenburg D. Jeninga E. H. Ruijtenbeek R. Kalkhoven E. (2009). Nuclear receptor-coregulator interaction profiling identifies TRIP3 as a novel peroxisome proliferator-activated receptor gamma cofactor. *Mol. Cell. Proteomics* 8 2212–2226. 10.1074/mcp.M900209-MCP200 19596656 PMC2758751

[B35] Lachize S. Apostolakis E. M. van der Laan S. Tijssen A. M. I. Xu J. de Kloet E. R. (2009). Steroid receptor coactivator-1 is necessary for regulation of corticotropin-releasing hormone by chronic stress and glucocorticoids. *Proc. Natl. Acad. Sci. U. S. A.* 106 8038–8042. 10.1073/pnas.0812062106 19416907 PMC2683087

[B36] Lewis-Tuffin L. J. Cidlowski J. A. (2006). The physiology of human glucocorticoid receptor beta (hGRbeta) and glucocorticoid resistance. *Ann. N. Y. Acad. Sci.* 1069 1–9. 10.1196/annals.1351.001 16855130

[B37] Loghmani H. Conway E. M. (2018). Exploring traditional and nontraditional roles for thrombomodulin. *Blood* 132 148–158. 10.1182/blood-2017-12-768994 29866818

[B38] Lu N. Z. Cidlowski J. A. (2005). Translational regulatory mechanisms generate N-terminal glucocorticoid receptor isoforms with unique transcriptional target genes. *Mol. Cell* 18 331–342. 10.1016/j.molcel.2005.03.025 15866175

[B39] Luecke H. F. Yamamoto K. R. (2005). The glucocorticoid receptor blocks P-TEFb recruitment by NFkappaB to effect promoter-specific transcriptional repression. *Genes Dev.* 19 1116–1127. 10.1101/gad.1297105 15879558 PMC1091745

[B40] Mani S. K. Allen J. M. Clark J. H. Blaustein J. D. O’Malley B. W. (1994). Convergent pathways for steroid hormone- and neurotransmitter-induced rat sexual behavior. *Science* 265 1246–1249. 10.1126/science.7915049 7915049

[B41] Matthews L. Berry A. Tersigni M. D’Acquisto F. Ianaro A. Ray D. (2009). Thiazolidinediones are partial agonists for the glucocorticoid receptor. *Endocrinology* 150 75–86. 10.1210/en.2008-0196 18801908 PMC4110506

[B42] McKenna N. J. O’Malley B. W. (2002). Combinatorial control of gene expression by nuclear receptors and coregulators. *Cell* 108 465–474. 10.1016/S0092-8674(02)00641-4 11909518

[B43] Meijer O. C. (2002). Coregulator proteins and corticosteroid action in the brain. *J. Neuroendocrinol.* 14 499–505. 10.1046/j.1365-2826.2002.00795.x 12047725

[B44] Meijer O. C. Kalkhoven E. van der Laan S. Steenbergen P. J. Houtman S. H. Dijkmans T. F. (2005). Steroid receptor coactivator-1 splice variants differentially affect corticosteroid receptor signaling. *Endocrinology* 146 1438–1448. 10.1210/en.2004-0411 15564339

[B45] Meijsing S. H. Pufall M. A. So A. Y. Bates D. L. Chen L. Yamamoto K. R. (2009). DNA binding site sequence directs glucocorticoid receptor structure and activity. *Science* 324 407–410. 10.1126/science.1164265 19372434 PMC2777810

[B46] Monczor F. Fernandez N. Legnazzi B. L. Riveiro M. E. Baldi A. Shayo C. (2003). Tiotidine, a histamine H2 receptor inverse agonist that binds with high affinity to an inactive G-protein-coupled form of the receptor. Experimental support for the cubic ternary complex model. *Mol. Pharmacol.* 64 512–520. 10.1124/mol.64.2.512 12869657

[B47] Moore J. M. R. Galicia S. J. McReynolds A. C. Nguyen N.-H. Scanlan T. S. Guy R. K. (2004). Quantitative proteomics of the thyroid hormone receptor-coregulator interactions. *J. Biol. Chem.* 279 27584–27590. 10.1074/jbc.M403453200 15100213

[B48] Newton R. Holden N. S. (2007). Separating transrepression and transactivation: a distressing divorce for the glucocorticoid receptor? *Mol. Pharmacol.* 72 799–809. 10.1124/mol.107.038794 17622575

[B49] Norman A. W. Mizwicki M. T. Norman D. P. G. (2004). Steroid-hormone rapid actions, membrane receptors and a conformational ensemble model. *Nat. Rev. Drug Discov.* 3 27–41. 10.1038/nrd1283 14708019

[B50] Oakley R. H. Cidlowski J. A. (2011). Cellular processing of the glucocorticoid receptor gene and protein: new mechanisms for generating tissue-specific actions of glucocorticoids. *J. Biol. Chem.* 286 3177–3184. 10.1074/jbc.R110.179325 21149445 PMC3030321

[B51] Ong K. M. Blackford J. A. Kagan B. L. Simons S. S. Chow C. C. (2010). A theoretical framework for gene induction and experimental comparisons. *Proc. Natl. Acad. Sci. U. S. A.* 107 7107–7112. 10.1073/pnas.0911095107 20351279 PMC2872427

[B52] Pfaff S. J. Fletterick R. J. (2010). Hormone binding and co-regulator binding to the glucocorticoid receptor are allosterically coupled. *J. Biol. Chem.* 285 15256–15267. 10.1074/jbc.M110.108118 20335180 PMC2865338

[B53] Pollard K. J. Peterson C. L. (1998). Chromatin remodeling: a marriage between two families? bioessays news. *Rev. Mol. Cell. Dev. Biol.* 20 771–780. 10.1002/(SICI)1521-1878(199809)20:9<771::AID-BIES10>3.0.CO;2-V 9819566

[B54] Power R. F. Mani S. K. Codina J. Conneely O. M. O’Malley B. W. (1991). Dopaminergic and ligand-independent activation of steroid hormone receptors. *Science* 254 1636–1639. 10.1126/science.1749936 1749936

[B55] Pujols L. Mullol J. Roca-Ferrer J. Torrego A. Xaubet A. Cidlowski J. A. (2002). Expression of glucocorticoid receptor alpha- and beta-isoforms in human cells and tissues. *Am. J. Physiol. Cell Physiol.* 283 C1324–C1331. 10.1152/ajpcell.00363.2001 12225995

[B56] Ratman D. Vanden Berghe W. Dejager L. Libert C. Tavernier J. Beck I. M. (2013). How glucocorticoid receptors modulate the activity of other transcription factors: a scope beyond tethering. *Mol. Cell. Endocrinol.* 380 41–54. 10.1016/j.mce.2012.12.014 23267834

[B57] Reddy T. E. Pauli F. Sprouse R. O. Neff N. F. Newberry K. M. Garabedian M. J. (2009). Genomic determination of the glucocorticoid response reveals unexpected mechanisms of gene regulation. *Genome Res.* 19 2163–2171. 10.1101/gr.097022.109 19801529 PMC2792167

[B58] Reul J. M. Hesketh S. A. Collins A. Mecinas M. G. (2009). Epigenetic mechanisms in the dentate gyrus act as a molecular switch in hippocampus-associated memory formation. *Epigenetics* 4 434–439. 10.4161/epi.4.7.9806 19829071

[B59] Rogatsky I. Wang J.-C. Derynck M. K. Nonaka D. F. Khodabakhsh D. B. Haqq C. M. (2003). Target-specific utilization of transcriptional regulatory surfaces by the glucocorticoid receptor. *Proc. Natl. Acad. Sci. U. S. A.* 100 13845–13850. 10.1073/pnas.2336092100 14617768 PMC283509

[B60] Ronchetti S. Migliorati G. Riccardi C. (2015). GILZ as a mediator of the anti-inflammatory effects of glucocorticoids. *Front. Endocrinol.* 6:170 10.3389/fendo.2015.00170 PMC4637413 26617572

[B61] Rousseau G. G. Baxter J. D. (1979). Glucocorticoid receptors. *Monogr. Endocrinol.* 12 49–77. 10.1007/978-3-642-81265-1_3 386089

[B62] Simons S. S. Chow C. C. (2012). The road less traveled: new views of steroid receptor action from the path of dose-response curves. *Mol. Cell. Endocrinol.* 348 373–382. 10.1016/j.mce.2011.05.030 21664235 PMC3184374

[B63] Siriani D. Mitsiou D. J. Alexis M. N. (2003). Overexpressed glucocorticoid receptor negatively regulates gene expression under conditions that favour accumulation of non-hormone-binding forms of the receptor. *J. Stero. Biochem. Mol. Biol.* 84 171–180. 10.1016/S0960-0760(03)00027-X 12711001

[B64] So A. Y.-L. Chaivorapol C. Bolton E. C. Li H. Yamamoto K. R. (2007). Determinants of cell- and gene-specific transcriptional regulation by the glucocorticoid receptor. *PLoS Genet.* 3:e94. 10.1371/journal.pgen.0030094 17559307 PMC1904358

[B65] So A. Y.-L. Cooper S. B. Feldman B. J. Manuchehri M. Yamamoto K. R. (2008). Conservation analysis predicts in vivo occupancy of glucocorticoid receptor-binding sequences at glucocorticoid-induced genes. *Proc. Natl. Acad. Sci. U. S. A.* 105 5745–5749. 10.1073/pnas.0801551105 18408151 PMC2311370

[B66] Stavreva D. A. Varticovski L. Hager G. L. (2012). Complex dynamics of transcription regulation. *Biochim. Biophys. Acta* 1819 657–666. 10.1016/j.bbagrm.2012.03.004 22484099 PMC3371156

[B67] Stavreva D. A. Wiench M. John S. Conway-Campbell B. L. McKenna M. A. Pooley J. R. (2009). Ultradian hormone stimulation induces glucocorticoid receptor-mediated pulses of gene transcription. *Nat. Cell Biol.* 11 1093–1102. 10.1038/ncb1922 19684579 PMC6711162

[B68] Tait A. S. Butts C. L. Sternberg E. M. (2008). The role of glucocorticoids and progestins in inflammatory, autoimmune, and infectious disease. *J. Leukoc. Biol.* 84 924–931. 10.1189/jlb.0208104 18664528 PMC2538604

[B69] Teichert A. Arnold L. A. Otieno S. Oda Y. Augustinaite I. Geistlinger T. R. (2009). Quantification of the vitamin D receptor-coregulator interaction. *Biochemistry* 48 1454–1461. 10.1021/bi801874n 19183053 PMC2654718

[B70] Tubio M. R. Fernandez N. Fitzsimons C. P. Copsel S. Santiago S. Shayo C. (2010). Expression of a G protein-coupled receptor (GPCR) leads to attenuation of signaling by other GPCRs: experimental evidence for a spontaneous GPCR constitutive inactive form. *J. Biol. Chem.* 285 14990–14998. 10.1074/jbc.M109.099689 20299453 PMC2865266

[B71] Voss T. C. John S. Hager G. L. (2006). Single-cell analysis of glucocorticoid receptor action reveals that stochastic post-chromatin association mechanisms regulate ligand-specific transcription. *Mol. Endocrinol.* 20 2641–2655. 10.1210/me.2006-0091 16873444

[B72] Wang J.-C. Derynck M. K. Nonaka D. F. Khodabakhsh D. B. Haqq C. Yamamoto K. R. (2004). Chromatin immunoprecipitation (ChIP) scanning identifies primary glucocorticoid receptor target genes. *Proc. Natl. Acad. Sci. U. S. A.* 101 15603–15608. 10.1073/pnas.0407008101 15501915 PMC524211

[B73] Wang Y. Ma N. Wang Y. Chen G. (2012). Allosteric analysis of glucocorticoid receptor-DNA interface induced by cyclic Py-Im polyamide: a molecular dynamics simulation study. *PloS One* 7:e35159. 10.1371/journal.pone.0035159 22532842 PMC3331974

[B74] Watson L. C. Kuchenbecker K. M. Schiller B. J. Gross J. D. Pufall M. A. Yamamoto K. R. (2013). The glucocorticoid receptor dimer interface allosterically transmits sequence-specific DNA signals. *Nat. Struct. Mol. Biol.* 20 876–883. 10.1038/nsmb.2595 23728292 PMC3702670

[B75] Weigel N. L. Zhang Y. (1998). Ligand-independent activation of steroid hormone receptors. *J. Mol. Med. Berl. Ger.* 76 469–479. 10.1007/s001090050241 9660165

[B76] Weiss J. M. Morgan P. H. Lutz M. W. Kenakin T. P. (1996). The cubic ternary complex receptor-occupancy model. III. *resurrecting efficacy*. *J. Theor. Biol.* 181 381–397. 10.1006/jtbi.1996.0139 8949584

[B77] Wu J. Li Y. Dietz J. Lala D. S. (2004). Repression of p65 transcriptional activation by the glucocorticoid receptor in the absence of receptor-coactivator interactions. *Mol. Endocrinol.* 18 53–62. 10.1210/me.2002-0373 14551261

[B78] Yamamoto K. R. Darimont B. D. Wagner R. L. Iñiguez-Lluhí J. A. (1998). Building transcriptional regulatory complexes: signals and surfaces. *Cold Spring Harb. Symp. Quant. Biol.* 63 587–598. 10.1101/sqb.1998.63.587 10384324

[B79] Yudt M. R. Jewell C. M. Bienstock R. J. Cidlowski J. A. (2003). Molecular origins for the dominant negative function of human glucocorticoid receptor beta. *Mol. Cell. Biol.* 23 4319–4330. 10.1128/MCB.23.12.4319-4330.2003 12773573 PMC156139

[B80] Zalachoras I. Houtman R. Atucha E. Devos R. Tijssen A. M. I. Hu P. (2013). Differential targeting of brain stress circuits with a selective glucocorticoid receptor modulator. *Proc. Natl. Acad. Sci. U. S. A.* 110 7910–7915. 10.1073/pnas.1219411110 23613579 PMC3651427

