# Supplementary material for: A Model of Glucocorticoid Receptor Interaction With Coregulators Predicts Transcriptional Regulation of Target Genes
Source: Front Pharmacol. 2019 Mar 13;10:214. doi: 10.3389/fphar.2019.00214 (PMC6425864; doi:10.3389/fphar.2019.00214)
Supplement: Supplementary file 1 [file Table_1.doc]

Supplementary Material

**A Model of Glucocorticoid Receptor Interaction with Coregulators Predicts Transcriptional Regulation of Target Genes.**

**Federico Monczor, Antonia Chatzopoulou, Carlos Daniel Zappia, René Houtman, Onno C. Meijer, Carlos P. Fitzsimons^*^**

* **Corresponding Author at:**

Neuroscience Program, Swammerdam Institute for Life Sciences, Faculty of Sciences, University of Amsterdam. SciencePark 904, 1098XH, Amsterdam, The Netherlands.

Email: [c.p.fitzsimons@uva.nl](mailto:c.p.fitzsimons@uva.nl)

**Supplementary Information 1**

The construction of our model is based the principle of microscopic balance (Wyman, 1975. The turning wheel: A study in steady states. Proc. natl. Acad. Sci. USA 72, 3983–3987), also known as the principle of microscopic reversibility (Lauffenburger & Linderman, 1993. Receptors: models for binding, trafficking, and signalling. New York: Oxford University Press). This principle is used to study reversible reactions once they achieve the equilibrium. The reactions considered in our model are state functions, depending only on the states of the system (namely initial and final), and thus do not depend on the [path](https://en.wikipedia.org/wiki/Thermodynamic_process_path) by which the system arrived at its present state. Thus, the system is primarily assumed in a dynamic equilibrium state. Three elemental equilibrium constants describe the binding of ligand, cofactors, and DNA to free glucocorticoid receptor (Ka, KC, and KD, respectively). These equilibrium constants can be altered by the presence of other or others partners previously bound to receptor. There are three parameters that modify the constants and describe how the binding of the ligand, cofactor, or DNA affects the binding of a second partner (alpha, beta and gamma), and a fourth parameter that characterize how the binding of any two partners affects the binding of the third. In all cases, when the parameter value is higher than 1, it means that the equilibrium is favored, reflecting a positive interaction. On the other hand, a value lesser than 1, reflects a negative interaction. The explanation and dependencies of the three constants and the four parameters are detailed on Supplementary Table S1.

In this context, considering that receptor response is proportional to the amount of receptor bound to cofactors and DNA (RCD and LRCD), an equation that to find its fractional magnitude respect to total receptor amount, can be obtained.

$\text{Response}=\frac{[RCD]+[LRCD]}{[R]+[RC]+[RD]+[LR]+[RCD]+[LRC]+[LRD]+[LRCD]}$ (Eq. 1)

If we represent the complex amount as a function of log [L], we can simulate a theoretical concentration-response assay for a given set of parameters. Taking into account Eq. 1 and considering the model-assumed equilibrium, its related constants,

$$\text{Ka}=\frac{[LR]}{[L]\cdot[R]}$$

$$\text{KC}=\frac{[RC]}{[R]\cdot[C]}$$

$$\text{KD}=\frac{[RD]}{[R]\cdot[D]}$$

and the conservation equation for the receptor,

$$R_{\text{total}}=R+RC+RD+LR+RCD+LRC+LRD+LRCD$$

we can deduce Eq. 2, which represents the variation in the concentration of the species assumed capable of generating a physiological response as a function of ligand concentration [L]

$$\text{Response}=\frac{\beta\cdot KC\cdot KD\cdot[C]\cdot[R_{total}]+\alpha\beta\gamma\delta\cdot Ka\cdot KC\cdot KD\cdot[C]\cdot[R_{total}]\cdot[L]}{1+KD+KC\cdot[C]+\beta\cdot KD\cdot KC\cdot[C]+(Ka+\gamma\cdot Ka\cdot KD+\alpha\cdot Ka\cdot KC\cdot[C]+\alpha\beta\gamma\delta\cdot Ka\cdot KC\cdot KD\cdot[C])\cdot[L]}$$

(Eq. 2)

Equation 2 describes a hyperbolic variation of response with L, with an upper asymptote (maximal response) when L tends to infinity

$$\text{Maximal Response}=\frac{\alpha\beta\gamma\delta\cdot KC\cdot KD\cdot[C]\cdot[R_{total}]}{1+\gamma\cdot KD+\alpha\cdot KC\cdot[C]+\alpha\beta\gamma\delta\cdot KC\cdot KD\cdot[C]}$$

a lower asymptote, when L is 0

$$\text{Basal Response}=\frac{\beta\cdot KC\cdot KD\cdot[C]\cdot[R_{total}]}{1+KD+KC\cdot[C]+\beta\cdot KD\cdot KC\cdot[C]}$$

and a middle point (EC_50_)

$$\text{EC}_{50}=\frac{\alpha\gamma\delta\cdot(1+KD+KC\cdot[C]+\beta\cdot KD\cdot KC\cdot[C])-2\cdot(1+\gamma\cdot KD+\alpha\cdot KC\cdot[C]+\alpha\beta\gamma\delta\cdot KD\cdot KC\cdot[C]}{\alpha\gamma\delta\cdot Ka\cdot(1+\gamma\cdot KD+(\alpha+\alpha\beta\gamma\delta\cdot KD)\cdot KC\cdot[C])}$$

If we represent *Response* as a function of log [L], we can simulate a theoretical dose-response assay for a given set of parameters (Fig. 3 of the main text).

Using this approach, a series of theoretical results can be predicted and experimentally tested in order to validate the model.

As a control of consistency, the prediction of the proportional diminishing of the maximal asymptote of the curves, and the no change of the middle point of the curve (EC50), when receptor number is decreased was subjected to experimental validation.

When GR expression was knocked down by means of siRNA cell transfection, the expected effects on the concentration-response curves was observed (Fig 4 of the main text).

Another feature of the model is the assumption that cofactors can bind the receptor even in the absence of DNA or ligand (RC species). This somehow conflicting behavior was tested using the MARCoNI technology approach. When the LBD domain of the glucocorticoid receptor was exposed to different amounts of several cofactors a binding of the receptor to cofactors pepetide was detected, and consistently in a cofactor amount-dependent manner (Fig 5 of the main text).

This result served as a basis to test cofactor binding to GR in different experimental conditions as described in the main text.

**Supplementary Table S1**

Description of the parameters used in the model. These are equivalent to ones described in the Cubic Ternary Complex Model of Receptor Occupancy.

| Parameter | Definition | Varies with... |
| --- | --- | --- |
| Ka | Equilibrium association constant for the binding of ligand to receptor. | Ligand, receptor |
| KD | Equilibrium association constant for the binding of receptor to DNA. | DNA, receptor |
| KC | Equilibrium association constant for the binding of coregulator to receptor. | Coregulator, receptor |
| α | Effect of ligand binding on the binding of coregulator, (or, alternatively, the effect of coregulator binding on ligand binding). α>1 means that the binding of ligand enhances the binding of coregulator to the receptor. | Ligand, coregulator, receptor |
| β | Effect of coregulator binding on the binding of receptor to DNA, (or, alternatively, the effect of binding of receptor to DNA on coregulator binding). β>1 means that the binding of coregulator enhances the binding of receptor to DNA. | Coregulator, DNA, receptor |
| γ | Effect of ligand binding on the binding of receptor to DNA, (or, alternatively, the effect of binding of receptor to DNA on ligand binding). γ>1 means that the binding of ligand enhances the binding of receptor to DNA. | Ligand, DNA, receptor |
| δ | Represents the extent to which the joint effect of any two of ligand binding, coregulator binding or DNA binding varies conditional on the level of the third. There are six different interpretations of δ. | Ligand, coregulator, DNA, receptor |

**Supplementary Table S2**.

NR Box peptides included in the PamGene chip used in this study. IDNR: identification number; Peptide name: coded as SwissProt Entry name_NR Box motif_motif position in the SwissProt aminoacid sequence_position of the first peptide aminoacid in the SwissProt sequence_position of the last peptide aminoacid in the SwissProt sequence_mutation present (optional); Gene name: official gene name approved by the HUGO gene nomenclature committee ([www.genenames.org](http://www.genenames.org/)); UniProtKB accession number: extracted from the UniprotKB/SwissProt database (http://www.uniprot.org/help/uniprotkb).

| **IDNR** | **Peptide name** | **Gene name** | **UniProtKB accession number** |
| --- | --- | --- | --- |
| 1 | CBP_LxxLL70_57_80 | CREBBP | Q92793 |
| 2 | CBP_LxxLL358_345_368_C367S | CREBBP | Q92793 |
| 3 | CBP_LxxLL358_345_368 | CREBBP | Q92793 |
| 4 | CBP_LxxLL2067_2055_2077 | CREBBP | Q92793 |
| 5 | DAX1_LxxML13_1_23 | NROB1 | P51843 |
| 6 | DAX1_LxxML80_68_90_C69S | NROB1 | P51843 |
| 7 | DAX1_LxxLL146_136_159 | NROB1 | P51843 |
| 8 | EP300_LxxLL81_69_91 | EP300 | Q09472 |
| 9 | EP300_LxxLL2051_2039_2061 | EP300 | Q09472 |
| 10 | IKBB_LxxLL74_62_84 | NFKBIB | Q15653 |
| 11 | IKBB_LxxLL289_277_299 | NFKBIB | Q15653 |
| 12 | JMJ1C_LxxLL2066_2054_2076 | JMJD1C | Q15652 |
| 13 | KIF11_LxxLL845_833_855_C855S | KIF11 | P52732 |
| 14 | NCOA1_LxxLL633_620_643 | NCOA1 | Q15788 |
| 15 | NCOA1_LxxLL690_677_700 | NCOA1 | Q15788 |
| 16 | NCOA1_LxxLL749_737_759 | NCOA1 | Q15788 |
| 17 | NCOA1_LxxLL1435_1421_1441 | NCOA1 | Q15788 |
| 19 | NCOA2_LxxLL641_628_651 | NCOA2 | Q15596 |
| 20 | NCOA2_LxxLL690_677_700 | NCOA2 | Q15596 |
| 21 | NCOA2_LxxLL745_733_755 | NCOA2 | Q15596 |
| 22 | NCOA2_LxxLL878_866_888 | NCOA2 | Q15596 |
| 23 | NCOA3_LxxLL113_102_123_N-KKK | NCOA3 | Q9Y6Q9 |
| 24 | NCOA3_LxxLL621_609_631 | NCOA3 | Q9Y6Q9 |
| 25 | NCOA3_LxxLL621_609_631_C627S | NCOA3 | Q9Y6Q9 |
| 26 | NCOA3_LxxLL685_673_695 | NCOA3 | Q9Y6Q9 |
| 27 | NCOA3_MOUSE_LxxLL1041_1029_1051 | Ncoa3 | O09000 |
| 28 | NCOA6_LxxLL887_875_897 | NCOA6 | Q14686 |
| 29 | NCOA6_LxxLL1491_1479_1501 | NCOA6 | Q14686 |
| 30 | NCOR1_LxxHI2051_2039_2061 | NCOR1 | O75376 |
| 31 | NCOR1_LxxHI2051_2039_2061_C2056S | NCOR1 | O75376 |
| 32 | NCOR1_LxxII2263_2251_2273 | NCOR1 | O75376 |
| 33 | NCOR2_LxxHI2135_2123_2145 | NCOR2 | Q9Y618 |
| 34 | NCOR2_LxxII2342_2330_2352 | NCOR2 | Q9Y618 |
| 35 | NRIP1_LxxLL133_121_143 | NRIP1 | P48552 |
| 36 | NRIP1_LxxLL185_173_195 | NRIP1 | P48552 |
| 37 | NRIP1_LxxLL185_173_195_C177S | NRIP1 | P48552 |
| 38 | NRIP1_LxxLL380_368_390 | NRIP1 | P48552 |
| 39 | NRIP1_LxxLL500_488_510 | NRIP1 | P48552 |
| 40 | NRIP1_LxxLL713_701_723 | NRIP1 | P48552 |
| 41 | NRIP1_LxxLL819_805_831 | NRIP1 | P48552 |
| 42 | NRIP1_LxxLL936_924_946 | NRIP1 | P48552 |
| 43 | NRIP1_LxxLL936_924_946_C945S | NRIP1 | P48552 |
| 44 | PCAF_LxxLL190_178_200 | KAT2B | Q92831 |
| 45 | PPRB_LxxLL604_591_614 | MED1 | Q15648 |
| 46 | PPRB_LxxLL645_632_655 | MED1 | Q15648 |
| 47 | PRGC1_LxxLL144_134_154 | PPARGC1A | Q9UBK2 |
| 48 | PRGC1_LxxLL144_130_155 | PPARGC1A | Q9UBK2 |
| 49 | PRGC2_LxxLL156_146_166 | PPARGC1B | Q86YN6 |
| 50 | PRGC2_LxxLL343_338_358 | PPARGC1B | Q86YN6 |
| 51 | SHP_LxxLL21_9_31_C9S_C11S | NR0B2 | Q15466 |
| 52 | SHP_LxxIL118_106_128 | NR0B2 | Q15466 |
| 53 | TRIP4_LxxLL161_149_171_C171S | TRIP4 | Q15650 |
| 54 | ZNHI3_LxxLL101_89_111 | ZNHIT3 | Q15649 |

**Supplementary Table S3**. 12 NR box peptides, which showed positive binding to the apo GR LBD. NR Box peptides names are coded as in Supplementary table S2.

| Peptide | Rmax | SEM | LogEC50 | SEM |
| --- | --- | --- | --- | --- |
| NRIP1_LxxLL185_173_195 | 1133 | 30.08 | -2.743 | 0.01072 |
| NRIP1_LxxLL21_8_30 | 5.236 | 5.362 | -4.504 | 0.2802 |
| NRIP1_LxxLL266_253_275_C263S | 35.7 | 8.103 | -2.73 | 0.1326 |
| NRIP1_LxxLL380_368_390 | 1196 | 69.93 | -2.763 | 0.03552 |
| NRIP1_LxxLL500_488_510 | 1164 | 278.1 | -2.371 | 0.1506 |
| NRIP1_LxxLL713_700_722 | 1000 | 898.9 | -2.238 | 0.9329 |
| NRIP1_LxxLL819_805_831 | 259.2 | 275.7 | -2.009 | 0.9554 |
| NRIP1_LxxLL936_924_946 | 2113 | 67.21 | -2.674 | 0.01457 |
| NRIP1_LxxML1068_1055_1077 | 1203 | 29.1 | -2.795 | 0.01248 |
| PRGC1_LxxLL144_130_155 | 42.16 | 2.113 | -4.628 | 0.1395 |
| PPRB_LxxLL645_632_655 | 576.2 | 72.14 | -2.685 | 0.07784 |
| ZNHI3_LxxLL101_89_111 | 1769 | 365.9 | -2.743 | 0.1382 |
